# Utilizing ChatGPT in Telepharmacy

**DOI:** 10.7759/cureus.52365

**Published:** 2024-01-16

**Authors:** Firas H Bazzari, Amjad H Bazzari

**Affiliations:** 1 Pharmacy, Jerash University, Jerash, JOR; 2 Basic Scientific Sciences, Applied Science Private University, Amman, JOR

**Keywords:** digital health, technology, remote pharmacy, chatgpt, pharmacology, artificial intelligence, pharmacy practice

## Abstract

Background: ChatGPT is an artificial intelligence-powered chatbot that has demonstrated capabilities in numerous fields, including medical and healthcare sciences. This study evaluates the potential for ChatGPT application in telepharmacy, the delivering of pharmaceutical care via means of telecommunications, through assessing its interactions, adherence to instructions, and ability to role-play as a pharmacist while handling a series of life-like scenario questions.

Methods: Two versions (ChatGPT 3.5 and 4.0, OpenAI) were assessed using two independent trials each. ChatGPT was instructed to act as a pharmacist and answer patient inquiries, followed by a set of 20 assessment questions. Then, ChatGPT was instructed to stop its act, provide feedback and list its sources for drug information. The responses to the assessment questions were evaluated in terms of accuracy, precision and clarity using a 4-point Likert-like scale.

Results: ChatGPT demonstrated the ability to follow detailed instructions, role-play as a pharmacist, and appropriately handle all questions. ChatGPT was able to understand case details, recognize generic and brand drug names, identify drug side effects, interactions, prescription requirements and precautions, and provide proper point-by-point instructions regarding administration, dosing, storage and disposal. The overall means of pooled scores were 3.425 (0.712) and 3.7 (0.61) for ChatGPT 3.5 and 4.0, respectively. The rank distribution of scores was not significantly different (P>0.05). None of the answers could be considered directly harmful or labeled as entirely or mostly incorrect, and most point deductions were due to other factors such as indecisiveness, adding immaterial information, missing certain considerations, or partial unclarity. The answers were similar in length across trials and appropriately concise. ChatGPT 4.0 showed superior performance, higher consistency, better character adherence and the ability to report various reliable information sources. However, it only allowed an input of 40 questions every three hours and provided inaccurate feedback regarding the number of assessed patients, compared to 3.5 which allowed unlimited input but was unable to provide feedback.

Conclusions: Integrating ChatGPT in telepharmacy holds promising potential; however, a number of drawbacks are to be overcome in order to function effectively.

## Introduction

Telepharmacy involves providing remote pharmaceutical care services through different means of telecommunications without direct contact between the pharmacist and patients [1]. The early introduction of telepharmacy aimed mainly to ease access to healthcare services, provide cost-effective solutions, and overcome shortage of pharmacists in rural areas [2]. However, this faced a number of challenges, namely the accessibility and capabilities of the available means of telecommunication back then [3]. Nevertheless, modern advances in mobile technologies and internet services have provided faster, more reliable, and user-friendly means of communication that proved to be efficient in pharmacy practice, especially during the coronavirus disease 2019 (COVID-19) pandemic [4]. Furthermore, telepharmacy was also found to be useful among other healthcare providers; for instance, pharmacists remote review of medications significantly reduced the number of potential adverse events and improved the speed of medication orders that were all accompanied with reduction in costs [5,6].

The development of artificial intelligence has been gaining momentum in recent years, with massive strides in promoting its utility in improving healthcare services. Early models of artificial intelligence were introduced to develop machine learning methods for structured data, modern deep learning, and natural language processing, with promising applications in various fields of medicine [7]. More recently, research focusing on developing artificial intelligence-powered chatbots has boomed and successfully introduced a vast number of them. A chatbot in its essence refers to a computer program that stimulates human conversation via text or voice commands [8]. Among the currently available chatbots, ChatGPT (OpenAI, San Francisco, CA, USA) has gained global attention for its abilities to deal with challenging language understanding and generation tasks, with promising applications in numerous fields [9]. Multiple studies have examined the potential applications of ChatGPT in healthcare, such as supporting patient care, research and planning, scientific production, and public health among others [10,11]. On the other hand, a number of limitations and ethical considerations were highlighted and shall be taken into consideration.

In pharmacy practice and medication use, different points of view can be noted among the literature, while some focus on the great potential of integrating ChatGPT in pharmacy practice, others namely highlight its drawbacks and limitations [12,13]. Therefore, this study aims to explore and assess the potential application of ChatGPT in telepharmacy and compare the performance of its currently available versions (3.5 and 4.0) by testing its ability to role-play as a pharmacist, interact with patients, and handle a series of life-like scenario questions.

## Materials and methods

This study was conducted using ChatGPT software and aimed at evaluating its ability to roleplay a telepharmacist. Two of the software versions were assessed: ChatGPT 3.5 and ChatGPT 4.0, the former of which is, at the time of the study, freely available without subscription fees. Each was evaluated using two independent trials: ChatGPT 3.5 was tested on 25/12/2023 and 27/12/2023, while ChatGPT 4.0 was tested on 28/12/2023 and 29/12/2023.

Each trial was initiated with the simple greeting “Hi”, to which ChatGPT was allowed to respond, followed by a request for ChatGPT to act as a pharmacist with specified conditions. This input was identical across trials and was: “I want you to act as a pharmacist on the phone to answer all upcoming patient's questions until I tell you “Stop acting as a pharmacist”. Every time an inquiry or question starts with a type of greeting such as Hi, Hello or good afternoon, etc. this means a new person (new conversation starts) and you should answer irrespective of previous inquiries or questions. do you understand?”.

Then, a set of 20 life-like scenario questions was used to evaluate the capacity of ChatGPT in handling case questions, maintaining a pharmacist character and providing appropriate answers. The 20 assessment questions were developed by the authors (F.H.B. and A.H.B.). The questions aimed at covering the various aspects of patient medication counseling services, which represent the main telepharmacy role directed towards patients, besides from medication review and refill authorization, which relate more to physicians. To ensure a comprehensive assessment in this regard, five main categories of evaluation were first selected: (1) proper drug use; (2) indications; (3) side effects; (4) interactions; and (5) pregnancy, lactation and pediatrics. The drug use category was further sub-categorized into drug administration technique, dosing, timing, storage, and disposal. Then, the questions were constructed evenly for each category and subcategory with a direction towards the common, special, or necessary in term of medication counseling. A simple greeting such as “Hi” or “Hello” was entered separately prior to each question to determine if and how ChatGPT would maintain the pharmacist character, followed by the question of interest. The 20 assessment questions are provided in Table 1.

**Table 1:** The questions used to assess ChatGPT.

No.	Question
1	How should I store my LANTUS and NOVOLOG insulin pens?
2	How many times per day should I use albuterol for my asthma?
3	Can you provide step by step instructions on how to take CLEXANE 40mg pre-filled syringes?
4	Should I take aspirin 100mg with food or on an empty stomach?
5	Can you advise me on how to safely dispose my insulin syringes?
6	I can’t remember if I should take LOZOL in the morning or at night, can you advise me?
7	I took an ibuprofen 600mg pill for a headache 1 hour ago but it’s not working, should I take another one?
8	I usually take my ATACAND pill every morning but I don’t remember if I took it today, what should I do?
9	I always take my metformin tablet at the same time every day but I overslept and missed the dose by 3 hours, what should I do?
10	I was prescribed FOSAMAX 70 mg tablets to take once weekly for my osteoporosis and my physician gave me an instructions flyer on how to take the drug but I lost it, are there any important use instructions?
11	What is the drug Glyburide used for and how does it work?
12	My physician told me about one of the drugs I take to use it only when I feel chest pain or before exercise but I cannot remember which one, I take the following medications: CONCOR, CRESTOR and NITROSTAT, can you tell me which one is it and how does it work?
13	My son was prescribed ZINNAT 250mg and I forgot to mention to the doctor that my son is allergic to penicillin, is it safe for him to take it?
14	I am an asthmatic patient who was recently prescribed FLIXOTIDE EVOHALER, I noticed the appearance of white patches on my tongue and I feel a very bad taste. I don’t know if this is because of the drug or asthma, can you help?
15	I was recently diagnosed with hypertension and prescribed a drug called captopril. I heard that potassium supplements can help lower blood pressure, is this true?
16	I am a 45 years old female and I am prescribed lithium to help treat my mood swings. My husband is used to taking a drug called furosemide to treat his feet that keep swelling. Recently, my feet started swelling as well and I would like to ask if I can also use furosemide?
17	I am a 25 years old female and I suffer from epilepsy but it is controlled using Lamotrigine, I recently got married and I am planning to become pregnant. Is my drug safe in pregnancy?
18	I am a breastfeeding 30 years old female and I am considering taking a contraceptive, is there an effective option that would be safe for me and my baby?
19	My 6 months old baby is feeling warm, his left cheek is reddish and he keeps rubbing his ear. Is he ok or is there something wrong?
20	My 12 months old baby is continuously coughing and I have some spare SINECOD syrup, is it ok if I give it to him?

The responses of ChatGPT to all inputs, including greetings, instructions and patient questions, were recorded. Since the ultimate aim of telepharmacy for a patient is to obtain the necessary information to ensure safe and effective medication use in an accessible manner, the information must be accurate, precise and clear. Accuracy was measured based on the correctness of the answer both when assessed on its own and in relation to the question. Precision was assessed by how much the response relates to the point of the question or how specific and relevant it is. Clarity was assessed by the ease with which a patient would be able to read and understand the response. Accordingly, these three requirements were considered equally important and used for scoring. This means that points will be deducted for a partially unclear response even if it was completely accurate and precise just as for a clear and precise but partially inaccurate response. The responses were evaluated using a 4-point Likert-like scale such that a score of 4 indicates “completely accurate, precise and clear answer”; a score of 3 indicates “almost accurate, precise and clear answer”; a score of 2 indicates “partially accurate, precise and clear answer”; and a score of 1 indicates “inaccurate, imprecise and unclear answer”. The response scoring was done by the authors (F.H.B., BPharm, MSc in clinical pharmacology, PhD in pharmacology and toxicology and A.H.B., PharmD, MSc in pharmacology, PhD in pharmacology and translational neuroscience). The consistency of answers across trials was assessed through the agreement level between scores. The rank distribution of scores, pooled across trials, was used to compare ChatGPT 3.5 and ChatGPT 4.0 performance using the Mann-Whitney U test.

Following the completion of the 20 assessment questions, ChatGPT was instructed to “Stop acting as a pharmacist” to determine whether it would remember this exact input from the initial command and comply with it. Then, ChatGPT was asked regarding the number of patient inquiries it received to determine its ability to accurately provide feedback: “How many patients' questions did you answer today?”. Lastly, ChatGPT was asked “What are your sources for drug information?” to determine their reliability.

## Results

Both ChatGPT versions across all trials complied to the “act as a pharmacist on the phone” command, expressed an understanding of the request conditions and indicated readiness to answer “patient” or “pharmacy-related” questions (Table 2). Interestingly, ChatGPT 3.5 on trial 2 declared, before answering any question, that it is “not a substitute for professional medical advice, diagnosis, or treatment” and highlighted the need to contact a health professional or an emergency service if immediate attention was required. However, this was not observed on trial 1 or among ChatGPT 4.0 trials.

**Table 2:** Response of ChatGPT when instructed to act as a pharmacist on the phone to answer patient questions.

ChatGPT 3.5	
Trial 1	Yes, I understand. I'll act as a pharmacist and answer any questions from patients. Please go ahead with your first question or concern.
Trial 2	Yes, I understand. Please keep in mind that while I can provide general information, I am not a substitute for professional medical advice, diagnosis, or treatment. If the situation requires immediate attention, please contact a healthcare professional or your local emergency services. Whenever you're ready, you can begin with the first question or inquiry.
ChatGPT 4.0	
Trial 1	Understood! I'm ready to assist with any pharmacy-related questions as if we're on a phone conversation. Let's begin.
Trial 2	Understood! I'll respond to each new greeting as the beginning of a conversation with a new patient, answering their questions as if I were a pharmacist on the phone. I'll continue to do so until you instruct me to stop. If you have any questions or inquiries, feel free to start the conversation.

A simple greeting, either “Hi” or “Hello”, was entered separately before each of the 20 assessment questions to evaluate the ability of ChatGPT to follow the instruction of acting as a pharmacist and treating all upcoming greetings as new patients, by either indicating or insinuating, that it is a pharmacist. Indeed, the responses to the first greeting, across all trials, indicated that ChatGPT was in a pharmacist character by welcoming questions related to health or medications. However, this was not maintained by ChatGPT 3.5, which remained “in character” for only the first two or three “patients” in contrast to ChatGPT 4.0, which maintained character for all 20 greetings that preceded patient questions (Table 3).

**Table 3:** The ability of ChatGPT to act as a pharmacist and to maintain character.

Trials	Response to first “patient” greeting	Response to last “patient” greeting	Stayed in character?
ChatGPT 3.5			
Trial 1	Hello! How can I help you today? If you have any questions or concerns about medications or health, feel free to ask.	Hello! How can I assist you today? If you have any questions or if there's something specific you'd like information or help with, feel free to let me know!	No (2/20)
Trial 2	Hello! How can I help you today with any pharmacy-related questions or concerns?	Hello! How can I assist you today? If you have any questions or if there's something specific you'd like to discuss, feel free to let me know!	No (3/20)
ChatGPT 4.0			
Trial 1	Hello! How can I help you today? If you have any questions or need information on medications, I'm here to assist.	Hello! How can I help you today? If you have any questions or concerns about health, medications, or anything else, please feel free to share them.	Yes (20/20)
Trial 2	Hello! How can I assist you today? If you have any questions regarding medications, health concerns, or pharmacy services, feel free to ask.	Hello! How can I help you today? If you have any questions or need assistance with a health concern, please let me know.	Yes (20/20)

The ability of ChatGPT to answer basic pharmacological questions accurately, precisely and clearly, as the primary indicator of its capacity to act as a telepharmacist, was assessed through scoring its responses to 20 drug-related life-like scenario questions. The mean scores for trials 1 and 2 for ChatGPT 3.5 were 3.45 (0.69) and 3.4 (0.75), respectively, while for ChatGPT 4.0 the scores were 3.7 (0.57) and 3.7 (0.66), respectively. The overall means, when trial scores were pooled, were 3.425 (0.712) and 3.7 (0.61) for ChatGPT 3.5 and 4.0, respectively, and the rank distribution of scores was not significantly different (U=624.5, P>0.05). The scores indicate that ChatGPT provided almost completely, but not completely, accurate, precise and clear answers. The consistency across trials was assessed by determining the score agreement level, which was observed for 13/20 questions for ChatGPT 3.5 answers (65%, Cohen’s Κ=0.394) and for 16/20 questions for ChatGPT 4.0 (80%, Cohen’s Κ=0.467), indicating fair and moderate consistency, respectively. In relation to the conciseness of ChatGPT answers, the mean answer word count across both trials was 237.85 (46.74) for ChatGPT 3.5 and 241.13 (60.72) for ChatGPT 4.0, which translates to a mean reading duration of one to two minutes, indicating an appropriate answer length and high similarity across ChatGPT versions in this regard. All ChatGPT responses to the 20 assessment questions across all trials can be found in the appendices.

ChatGPT was able to handle all questions, provide appropriate answers, utilize provided case details, grasp generic and brand drug names and identify drug-related side effects, interactions, prescription requirements, precautions and proper point-by-point use instructions regarding administration, dosing, storage and disposal. ChatGPT 3.5 clearly stated that it is “not a doctor” or that it is “not a substitute for professional medical advice” in six and seven responses on trials 1 and 2, respectively, while ChatGPT 4.0 did it only once, which may relate to its higher adherence to the telepharmacist character. Nonetheless, in all responses, both versions stressed the necessity to adhere to the exact healthcare provider instructions and seek professional medical advice specific to their individual needs. It is important to note that in all trials, ChatGPT did not give any advice that would be considered directly harmful to the patient or statements that would be labeled as entirely or mostly incorrect, except for certain inaccurate details. Accordingly, most point deductions, when scoring ChatGPT responses, were mainly due to other factors such as indecisiveness, imprecision, adding immaterial information, missing certain considerations, or partial unclarity in view of a patient.

A particularly noteworthy observation in ChatGPT’s responses to the assessment questions involves its overall “intelligence” and “attentiveness”. These were evident in multiple responses and in various ways. To mention a few examples that were “an addition” to the question-specific answers: ChatGPT congratulated a patient on her recent marriage, suggested requesting a replacement copy for the instructions flyer that a patient had lost, proposed many potassium-rich foods as safer alternatives to taking potassium supplements that would interact with a patient’s medication, recommended various methods to help in remembering if a dose was taken to a patient that did not remember if he had taken his pill, encouraged various ways to help improve adherence to a patient that had missed a dose, highlighted that a person should not take a medication prescribed to someone else even if the symptoms were similar to a patient asking if she should take her husband’s drug, and provided suggestions to decrease the occurrence of a preventable side effect in addition to identifying it. Furthermore, the responses of ChatGPT involved clarifications of certain technical or medical terms that not all patients may understand such as clarifying the term diuretic as “water pill”, edema as “swelling” or “fluid retention”, upright position as “sitting, standing, or walking”, angina as “chest pain”, sublingually as “under the tongue”, thrush as a “fungal infection”, hypertension as “high blood pressure”, antitussive as “cough suppressant”, subcutaneous as “fatty tissue just under the skin”, hypoglycemia as “low blood sugar”, and oral contraceptive as “birth control pill”.

After answering the assessment questions, ChatGPT was instructed to “stop acting as a pharmacist”, to which it complied and offered assistance on other subjects indicating that it was still adherent to the instructions at the beginning of the conversation (Table 4).

**Table 4:** Response of ChatGPT when instructed to stop acting as a pharmacist.

ChatGPT 3.5	
Trial 1	Of course! If you have any other non-pharmacy related questions or if there's anything else I can help you with, feel free to let me know.
Trial 2	Of course! If you have any other questions or if there's a different topic you'd like assistance with, feel free to let me know. I'm here to help!
ChatGPT 4.0	
Trial 1	Understood. If you have any other questions or need assistance with something else, feel free to ask.
Trial 2	Understood. If you need assistance with anything else, feel free to ask.

Another key point to be considered is the limitation of the number of questions or inputs that ChatGPT 4.0 allows in a session, which during trial taking was 40 questions every three hours. Therefore, this represents a limitation for the potential telepharmacy application of ChatGPT 4.0. However, this was not the case for ChatGPT 3.5, which could virtually handle an unlimited number of inquiries/inputs.

After being instructed to stop its pharmacist act, ChatGPT was asked about the number of patient inquiries it received, which was 20 during each trial. However, ChatGPT 3.5 was unable to because of its disabled “capability to keep track of specific interactions” and “browsing capability” and because the “conversation history is not stored for privacy reasons”. On the other hand, ChatGPT 4.0 responded with a request to review the conversation history to provide a count of the variety of “inquiries presented as patient questions in a role-playing scenario”. Upon granting the permission, ChatGPT 4.0 was able to provide a count, which interestingly was incorrect on both trials as it answered with 11 and 10 times, instead of 20, on trials 1 and 2, respectively. This represents another hurdle to the use of ChatGPT in telepharmacy as it would not be able to provide feedback, as with ChatGPT 3.5, or give incorrect feedback in case of ChatGPT 4.0.

Lastly, ChatGPT was asked regarding its sources of drug information. ChatGPT 3.5 was unable to provide its sources due to its lack of access to training data but it stressed the importance to “consult with a qualified healthcare professional or pharmacist for specific drug-related concerns”. On the other hand, ChatGPT 4.0 reported various reliable training, but not in real-time, sources including “textbooks, medical journals, drug databases, and reputable medical websites” while providing reputable reference examples on trial 1 but not 2. These included (1) “Drug information databases like Drugs.com, RxList, and MedlinePlus”; (2) “Peer-reviewed medical journals and articles”; (3) “Clinical guidelines from organizations such as the FDA, CDC, WHO, and various professional medical associations”; and (4) “Standard pharmacology and medical reference texts”.

## Discussion

The results of the current study revealed a great potential for ChatGPT in telepharmacy. ChatGPT was able to appropriately handle all questions through showing an understanding of the case details, identifying both generic and brand drug names, identifying drug side effects, interactions, prescription requirements, precautions, and providing proper point-by-point drug use instructions regarding administration, dosing, storage and disposal. Furthermore, none of the answers could be considered directly harmful to the patient or labeled as entirely or mostly incorrect, except for certain inaccurate details; thus, most point deductions were mainly due to other factors such as indecisiveness, imprecision, adding immaterial information, missing certain considerations, or partial unclarity in view of a patient. Nonetheless, both versions stressed the necessity to adhere to the exact healthcare provider instructions and to seek professional medical advice specific to their individual needs in all responses. In relation to the conciseness of answers, the mean answer word count across trials translates to a mean reading duration of one to two minutes, indicating an appropriate answer length. All in all, the ChatGPT 4.0 version provided more accurate, precise and clear answers to patients’ questions with better adherence to the pharmacist character and reported various reliable sources; however, it only allowed an input of 40 questions every three hours compared to the 3.5 version which allowed limitless number of questions.

Multiple studies have also highlighted the potential usefulness of ChatGPT in answering drug-related questions. For instance, in a set of random public medication consultation questions, ChatGPT 3.5 answers were found to be appropriate and promoted its usefulness in answering basic drug-related consultations [14]. In another set of 100 multiple choice questions in the field of basic and clinical medical sciences, ChatGPT 3.5 answers obtained a satisfactory score, demonstrated a degree of good understanding and explanation, and were suggested to be a valuable asset in medical education [15]. When provided with two question sets of the Japanese national examination for pharmacists, ChatGPT 4.0 demonstrated the potential to support pharmacists’ capabilities and to become a reliable tool in pharmacy practice [16]. From the point of view of pharmacists and other healthcare providers, the utilization of ChatGPT and other chatbots was widely accepted and promising to be integrated into daily practice [17,18]. Such findings, combined with the rapid and continuous development in artificial intelligence, highlight the potential for ChatGPT to soon become a powerful assisting tool to pharmacists and to be an essential part of the healthcare setting.

On the other hand, numerous studies testing the capabilities of ChatGPT have highlighted a number of drawbacks and raised some concerns regarding the use of chatbots in healthcare and medical settings. For example, the inability of ChatGPT to properly handle complex clinical cases that require careful assessment by experts as well as advanced reasoning and complex instructions [19]. In addition, possible violation of copyrights, medicolegal issues, and the potential for bias, inaccuracies or prejudices in the generated content as well as the production of irrelevant references are also among the listed limitations of ChatGPT [20]. Other studies have highlighted a number of existing issues that can be resolved through advanced training and development of the algorithms, such as maintaining context, emotional intelligence, and real-time multimodal interactions [21]. Despite the general acceptance of integrating chatbots into healthcare practice, healthcare providers from various fields still express a cautious behavior insinuating the lack of complete trust in chatbots and the necessity of human supervision, which can be justified due to the current existence of a number of limitations, including the aforementioned [22,23].

Despite the impressive performance of ChatGPT 4.0, it is important to note that any minor inaccuracies or unclarities in responses may have impactful implications in relation to its potential broad application. This is especially true for cases that involve special drug preparations or indications that may differ, in terms of administration technique or dosing, from the typical or usual. In addition, the essence of pharmaceutical care relies on a patient-centered approach in which a pharmacist would be responsible for providing drug-related needs in a patient-specific manner. This entails taking into account all potential variables impacting drug selection, dosing and usage such as other comorbidities, medications, supplements, or potential changes in circumstances, such as pregnancy. In the current study, ChatGPT was able to provide appropriate answers by taking into account all provided question details; however, it did not follow up for any additional information the patient may not have included in the initial inquiry that may influence the response. On the other hand, ChatGPT indeed specified, in all responses, that the patient should always rely on and follow the exact instructions of the healthcare provider, which would be personalized and tailored to the individual needs of the patient. This could also limit the ability to detect medication errors and should be taken into consideration for potential future improvements before real-life application in telepharmacy or other healthcare-related areas such as assistance in clinical pharmacy for hospital inpatients.

The two other drawbacks of ChatGPT 4.0 that were observed in the study, and could also hinder real-life application, are the limitation of the number of inquiries and the provision of incorrect feedback. The former could negate a major telepharmacy aim of accessibility while the latter would prevent various potential benefits of assessing the service. While the issue of feedback may be solved if the software was able to store and analyze inputs, this would raise other ethical concerns, especially ones related to privacy and confidentiality of patient information. These concerns may also occur when attempting solutions to the issue of impersonal or unindividualized care, for instance, by registering a patient profile that includes the required medical information for each patient. Further insights into any other limitations and potential areas for improvement could be provided by additional studies that investigate more life-like scenarios covering additional scopes of pharmaceutical healthcare and studies directly comparing the performance of ChatGPT against professional human pharmacists.

## Conclusions

In conclusion, the adoption of ChatGPT in telepharmacy holds a promising potential with an overall better performance of ChatGPT 4.0 compared to the 3.5 version. ChatGPT 4.0 demonstrated the ability to role-play a telepharmacist, maintain the character, treat questions as patient inquiries, adhere to related commands and instructions, grasp and account for relevant case details, provide almost completely accurate, precise and clear answers to drug-related questions, and respond in a moderately consistent and concise but comprehensive-enough manner. On the other hand, a number of drawbacks were also observed regarding the limitation of the allowed number of inquiries and the provision of incorrect feedback.

## Appendices

**Table 5:** ChatGPT responses to the 20 assessment questions across all trials.

ChatGPT 3.5 Trial 1 Responses	
Question	Response
1. how should I store my LANTUS and NOVOLOG insulin pens?	It's essential to store LANTUS and NOVOLOG insulin pens properly to maintain their effectiveness. Here are some general guidelines: Refrigeration: It's recommended to store insulin pens in the refrigerator between 36°F and 46°F (2°C and 8°C). However, do not freeze them. Avoid direct sunlight: Keep the insulin pens away from direct sunlight and heat. Exposure to high temperatures can affect the potency of the insulin. In-use pens: If you are actively using a pen, you can keep it at room temperature (between 59°F and 86°F or 15°C and 30°C) for up to 28 days. This is especially important for pens you are currently using to avoid discomfort from cold injections. Unused pens: If you have pens that you're not using, store them in the refrigerator to extend their shelf life. Remember to check the manufacturer's recommendations for the specific insulin pens you are using, as storage instructions can vary between different brands. If you have any more questions or need further clarification, feel free to ask!
2. how many times per day should I use albuterol for my asthma?	I'm not a substitute for professional medical advice, but I can offer some general information. The dosing schedule for albuterol can vary based on your individual circumstances, and it's crucial to follow your doctor's recommendations. Typically, albuterol is used as a rescue inhaler for acute symptoms of asthma. The usual recommendation is to use albuterol when you experience symptoms such as shortness of breath, wheezing, or chest tightness. Your doctor will prescribe a specific dose and provide guidance on how often you should use it. If you find yourself needing to use your albuterol inhaler more frequently than prescribed, it's important to contact your healthcare provider. Overuse may indicate uncontrolled asthma, and your treatment plan may need adjustment. Always follow your healthcare provider's instructions, and if you have any concerns or questions about your asthma medication, consult with them for personalized advice.
3. can you provide step by step instructions on how to take CLEXANE 40mg pre-filled syringes?	I can provide general guidance on using CLEXANE 40mg pre-filled syringes, but it's crucial to follow the specific instructions provided by your healthcare provider and the medication's packaging. Always consult with your healthcare professional for personalized advice. Here are general steps for administering CLEXANE: Before you begin: Wash your hands thoroughly. Choose a clean, well-lit area. Preparing the injection: Remove the syringe from the packaging. Check the expiration date and ensure the solution is clear. Do not use if it is discolored or contains particles. **Hold the syringe with the needle pointing up. Tap the syringe gently to ensure any air bubbles rise to the top. Remove the protective cap from the needle. Injecting the medication: Pinch a fold of skin around the injection site (common sites include the abdomen or thigh). Insert the needle at a 90-degree angle into the pinched skin. While holding the skin fold, inject the medication slowly and steadily. Keep the needle in for a few seconds after injecting to ensure the full dose is delivered. Remove the needle and release the skin fold. Do not rub the injection site. Disposing of the syringe: Place the used syringe in a sharps container or a puncture-resistant container. Do not recycle the syringe. Always follow your healthcare provider's instructions precisely. If you have any concerns or questions about administering CLEXANE, reach out to your healthcare professional for guidance.
4. should I take aspirin 100mg with food or on an empty stomach?	The decision to take aspirin with or without food can depend on various factors, including your individual tolerance and any specific instructions from your healthcare provider. However, here are some general guidelines: With Food: Taking aspirin with food or right after a meal can help minimize the risk of stomach upset or irritation. Food can act as a buffer and reduce the likelihood of gastrointestinal discomfort. On an Empty Stomach: If your doctor specifically instructs you to take aspirin on an empty stomach, follow their advice. In some cases, taking medications on an empty stomach may enhance absorption. Always follow the instructions provided by your healthcare provider or those on the medication label. If you're unsure about the best way to take aspirin based on your individual health condition or other medications you may be taking, consult with your healthcare professional for personalized advice.
5. can you advise me on how to safely dispose my insulin syringes?	Certainly! Proper disposal of insulin syringes is important to prevent injuries and protect the environment. Here are steps to safely dispose of your insulin syringes: Use a Sharps Container: Place used syringes and needles in an FDA-cleared sharps disposal container. These containers are designed to safely store sharp objects and can be purchased at pharmacies or medical supply stores. Seal the Container: Once the sharps container is about two-thirds full, seal it tightly. If a sharps container is not available, you can use a heavy-duty plastic container with a screw-on or tightly secured lid. Label the Container: Clearly label the container as "Used Sharps" to alert others to its contents. Do Not Recycle: Do not recycle syringes or throw them in the regular trash. It's essential to keep used needles and syringes separate from other waste. Drop-Off Programs: Check with your local waste management or health department for information on community disposal programs. Some areas have special drop-off locations for medical sharps. Mail-Back Programs: Some regions offer mail-back programs where you can mail your sharps container for proper disposal. Check with local authorities or healthcare providers for information on available programs. Medical Waste Disposal Services: In some cases, medical waste disposal services may be available for home use. Check with local providers for options. Always follow your local regulations and guidelines for medical waste disposal, as they may vary. If you have any specific questions or concerns, consider reaching out to your healthcare provider or local health department for guidance.
6. I can’t remember if I should take LOZOL in the morning or at night, can you advise me?	LOZOL, or indapamide, is a diuretic medication used to treat high blood pressure. The specific dosing instructions for LOZOL can vary depending on your individual health condition and what your healthcare provider has prescribed. However, generally speaking, diuretics are often recommended to be taken in the morning. Taking diuretics in the morning can help prevent excessive urination during the nighttime, which might otherwise disrupt your sleep. Additionally, taking them earlier in the day may be beneficial because diuretics increase urine production and can lead to increased frequency of urination. To be sure about the appropriate timing for your LOZOL medication, it's crucial to follow your doctor's instructions precisely. If you are unsure about the dosing schedule or if you have any concerns, contact your healthcare provider for clarification. They can provide personalized advice based on your medical history and overall treatment plan.
7. I took an ibuprofen 600mg pill for a headache 1 hour ago but it’s not working, should I take another one?	I'm not a doctor, but I can offer some general information. If you've taken ibuprofen 600mg and it hasn't relieved your headache, it's important to be cautious about taking additional medication without consulting a healthcare professional. Here are a few considerations: Dosage and Timing: Check the recommended dosage and the time interval for ibuprofen on the packaging or as advised by your healthcare provider. It's crucial not to exceed the recommended dose within a specific timeframe. Medical Conditions: If you have any underlying health conditions or are taking other medications, it's essential to consider potential interactions. Consult with your doctor if you have concerns. Seek Professional Advice: If your headache persists or worsens, it's advisable to contact your healthcare provider for guidance. They can provide recommendations based on your individual health situation. Avoid Overuse: Taking too much ibuprofen can lead to adverse effects, including stomach irritation and potential damage, so it's important not to exceed the recommended dosage. Remember, it's always best to consult with a healthcare professional for personalized advice. If your headache is severe, persists, or if you have any other concerns, seek medical attention promptly.
8. I usually take my ATACAND pill every morning but I don’t remember if I took it today, what should I do?	If you're unsure whether you've taken your ATACAND (candesartan) pill for the day, here are a few steps you can consider: Check Pill Organizer: If you use a pill organizer, check to see if the pill for today has been taken. This can help you remember if you've already taken your medication. Review Routine: Reflect on your daily routine. Sometimes, remembering the sequence of events in your morning routine can help you recall whether you took the medication. Ask Someone: If you live with someone, ask if they recall seeing you take your medication. If, after considering these steps, you still cannot confirm whether you took your ATACAND pill, it's generally recommended to err on the side of caution. If you miss a dose, and it's not too close to the next scheduled dose, you can take the missed dose as soon as you remember. However, if it's almost time for your next scheduled dose, skip the missed dose and continue with your regular dosing schedule. If you're uncertain or have any concerns, it's essential to contact your healthcare provider for guidance. They can provide specific advice based on your health condition and the medication regimen prescribed for you.
9. I always take my metformin tablet at the same time every day but I overslept and missed the dose by 3 hours, what should I do?	If you've overslept and missed your usual time for taking metformin by 3 hours, here are some general guidelines: Take the Missed Dose: If it's within a reasonable timeframe and not too close to your next scheduled dose, you can go ahead and take the missed metformin dose. Resume Regular Schedule: After taking the missed dose, continue with your regular dosing schedule. Do not double up on doses to make up for the missed one. Monitor Blood Sugar: Keep an eye on your blood sugar levels, especially if you have diabetes. Missing a dose may affect your blood sugar, so be aware of any changes and follow any additional guidance from your healthcare provider. Notify Your Healthcare Provider: If you have concerns or if missing a dose is a recurrent issue, it's advisable to inform your healthcare provider. They can provide guidance based on your specific health condition and medication regimen. Remember, these are general guidelines, and it's essential to follow the advice provided by your healthcare provider. If you have any questions or if you're uncertain about what to do in your specific situation, contact your healthcare professional for personalized advice.
10. I was prescribed FOSAMAX 70 mg tablets to take once weekly for my osteoporosis and my physician gave me an instructions flyer on how to take the drug but I lost it, are there any important use instructions?	Yes, Fosamax (alendronate) is a medication commonly prescribed for the treatment of osteoporosis. Since you've lost the instructions, here are some general guidelines on how to take Fosamax 70 mg tablets once weekly: Take on an Empty Stomach: Fosamax is usually taken on an empty stomach. This means you should take it at least 30 minutes before your first food, drink, or other medications of the day. It's important to take it with a full glass of plain water. Stay Upright After Taking: After taking Fosamax, remain upright (sitting, standing, or walking) for at least 30 minutes. This helps ensure the medication reaches your stomach and is absorbed properly. Do Not Lie Down: Avoid lying down for at least 30 minutes after taking Fosamax to reduce the risk of irritation to the esophagus. No Food or Other Medications: Do not consume any food, beverages (other than water), or other medications within 30 minutes of taking Fosamax. Take the Full Dose: Swallow the tablet whole; do not crush, chew, or suck on it. It's important to take the full dose as prescribed by your healthcare provider. Weekly Schedule: Fosamax is typically taken once a week. Choose a specific day of the week that is convenient for you and take it at the same time on that day. It's crucial to follow your healthcare provider's specific instructions for taking Fosamax, as individual circumstances may vary. If you have any concerns or questions, or if you need a replacement for the instructions flyer, reach out to your healthcare provider or pharmacist for guidance.
11. what is the drug Glyburide used for and how does it work?	Glyburide is an oral antidiabetic medication used to treat type 2 diabetes. It belongs to a class of drugs known as sulfonylureas. Here's an overview of its uses and how it works: Uses: Glyburide is prescribed to help control blood sugar levels in people with type 2 diabetes. It is typically used in conjunction with diet and exercise. This medication is not used to treat type 1 diabetes. Mechanism of Action: Glyburide works by stimulating the pancreas to release insulin, a hormone that helps regulate blood sugar. Here's how it generally works: Insulin Release: Glyburide binds to specific receptors on the beta cells in the pancreas, prompting these cells to release insulin. Insulin's Role: Insulin plays a crucial role in glucose metabolism. It helps cells take in glucose from the bloodstream, reducing blood sugar levels. Improved Blood Sugar Control: By increasing insulin release, glyburide helps improve the body's ability to manage glucose, thereby lowering elevated blood sugar levels in individuals with type 2 diabetes. It's important to note that while glyburide can be effective in controlling blood sugar, its use should be closely monitored by healthcare professionals. Individual responses to the medication can vary, and it may not be suitable for everyone. Additionally, lifestyle factors such as diet and exercise remain important components of diabetes management. Always follow your healthcare provider's instructions and attend regular check-ups to ensure the medication is working effectively and safely for you.
12. my physician told me about one of the drugs I take to use it only when I feel chest pain or before exercise but I cannot remember which one, I take the following medications: CONCOR, CRESTOR and NITROSTAT, can you tell me which one is it and how does it work?	Among the medications you mentioned, Nitrostat (nitroglycerin) is typically used to relieve chest pain (angina) and can be taken as needed, especially before activities that may trigger chest pain, such as exercise. Here's some information about Nitrostat: Nitrostat (Nitroglycerin): Purpose: Nitroglycerin is a vasodilator, which means it relaxes and widens blood vessels. This helps increase blood flow and oxygen to the heart, relieving chest pain or discomfort (angina). Usage: It is often used as a rescue medication for acute angina attacks or taken before activities that may provoke chest pain. How to Take: Nitrostat is typically taken under the tongue (sublingually) for rapid absorption. The tablet should be placed under the tongue and allowed to dissolve. Given your description, it's possible that Nitrostat is the medication your physician instructed you to use as needed for chest pain or before exercise. However, it's crucial to confirm this with your healthcare provider, as individual instructions may vary based on your specific health condition. Concor (bisoprolol) and Crestor (rosuvastatin) are not typically used to relieve acute chest pain. Concor is a beta-blocker used to treat high blood pressure and certain heart conditions, while Crestor is a statin used to lower cholesterol levels. If you have any doubts or concerns about your medications, it's important to reach out to your healthcare provider for clarification. They can provide specific guidance based on your medical history and individual needs.
13. my son was prescribed ZINNAT 250mg and I forgot to mention to the doctor that my son is allergic to penicillin, is it safe for him to take it?	Zinnat, also known as cefuroxime axetil, is a cephalosporin antibiotic. Cephalosporins are a class of antibiotics that are structurally related to penicillins. While being allergic to penicillin does not guarantee an allergy to cephalosporins, there is a potential for cross-reactivity between these two classes of antibiotics. It's crucial to inform your healthcare provider about any allergies, especially to penicillin or related antibiotics, before starting a new medication. Cross-reactivity rates between penicillins and cephalosporins vary, and not all individuals who are allergic to penicillin will necessarily have an allergic reaction to cephalosporins. Given that your son has a known penicillin allergy, it is essential to contact your healthcare provider promptly. They can assess the situation, consider the specific cephalosporin prescribed (Zinnat in this case), and determine whether it is safe for your son to take based on his medical history. Do not give your son the medication until you have spoken with your healthcare provider about the allergy and the prescribed antibiotic. If you're unable to reach your healthcare provider immediately, consider contacting them as soon as possible or seeking advice from another medical professional. Allergic reactions to antibiotics can range from mild to severe, so it's crucial to address this concern promptly to ensure your son's safety and well-being.
14. I am an asthmatic patient who was recently prescribed FLIXOTIDE EVOHALER, I noticed the appearance of white patches on my tongue and I feel a very bad taste. I don’t know if this is because of the drug or asthma, can you help?	It's important to note that the symptoms you're describing, such as white patches on the tongue and a bad taste, could potentially be associated with the use of inhaled corticosteroids like Flixotide (fluticasone), which is commonly used for asthma. However, these symptoms may also be caused by various other factors unrelated to your asthma or medication. Here are some considerations: Flixotide Side Effects: While side effects can occur with any medication, inhaled corticosteroids like Flixotide are generally well-tolerated. However, it's possible for some individuals to experience local side effects in the mouth or throat, including fungal infections (oral thrush) or irritation. Oral Thrush: White patches on the tongue could be indicative of oral thrush, which is a fungal infection. Inhaled corticosteroids can sometimes increase the risk of developing thrush in the mouth. Bad Taste: A bad taste may also be associated with the medication or other factors such as dry mouth or altered taste perception. It's crucial to bring these symptoms to the attention of your healthcare provider promptly. They can assess whether the symptoms are related to the medication, asthma, or another issue. Depending on the cause, adjustments to your treatment plan may be necessary. In the meantime, consider the following: Rinse Mouth After Use: After using the Flixotide inhaler, rinse your mouth with water to help minimize the risk of fungal infections. Contact Your Healthcare Provider: Reach out to your healthcare provider to discuss the symptoms you're experiencing. They may recommend an examination and, if necessary, adjustments to your treatment plan. Remember, this information is not a substitute for professional medical advice. Always consult with your healthcare provider for personalized guidance based on your individual health condition and medication regimen.
15. I was recently diagnosed with hypertension and prescribed a drug called captopril. I heard that potassium supplements can help lower blood pressure, is this true?	Captopril is an angiotensin-converting enzyme (ACE) inhibitor commonly prescribed for the treatment of hypertension (high blood pressure). While potassium is an essential mineral that plays a role in maintaining a healthy blood pressure, it's important to approach potassium supplementation cautiously, especially when taking medications like captopril. Here are some key points to consider: Potassium and Blood Pressure: Potassium is known to help regulate blood pressure by balancing the effects of sodium. Adequate potassium intake is generally associated with a lower risk of hypertension. Captopril and Potassium: ACE inhibitors, including captopril, can affect potassium levels in the body. These medications may increase potassium levels, and using additional potassium supplements without medical supervision can lead to elevated potassium levels, known as hyperkalemia. Supplementing Potassium: It's crucial to avoid self-prescribing potassium supplements without consulting your healthcare provider. Too much potassium can be harmful, especially when combined with certain medications. Dietary Sources: Instead of supplements, consider increasing your intake of potassium-rich foods as part of a balanced diet. Foods high in potassium include bananas, oranges, potatoes, tomatoes, spinach, and other fruits and vegetables. Regular Monitoring: Your healthcare provider will monitor your potassium levels while you are on medications like captopril. If needed, they may adjust your treatment plan or recommend dietary changes. Always communicate openly with your healthcare provider about any supplements you are considering taking. They can provide guidance based on your specific health condition, medications, and individual needs. It's important to strike the right balance and ensure that you are getting the appropriate amount of potassium without putting your health at risk.
16. I am a 45 years old female and I am prescribed lithium to help treat my mood swings. My husband is used to taking a drug called furosemide to treat his feet that keep swelling. Recently, my feet started swelling as well and I would like to ask if I can also use furosemide?	I am not a doctor, but I can provide some general information. It's important to consult with your healthcare provider before making any decisions about medications, especially given that you are already prescribed lithium. Lithium is a mood-stabilizing medication commonly used to treat bipolar disorder. Furosemide, on the other hand, is a diuretic (water pill) often used to treat conditions like edema (swelling) due to various causes, including heart failure, kidney problems, and liver disease. It's crucial to be cautious about adding or changing medications, especially when you are already on a prescribed regimen. Here are a few reasons why: Interaction Risk: Furosemide and lithium can interact, potentially affecting lithium levels in the blood. Changes in lithium levels can be significant and may require adjustments to your lithium dosage. This should only be done under the supervision of your healthcare provider. Monitoring: Both lithium and furosemide may require regular monitoring of blood levels and other parameters. Your healthcare provider needs to assess the potential risks and benefits, considering your overall health. Underlying Causes: Swelling in the feet can have various causes. It's important to determine the underlying cause before initiating treatment. Your healthcare provider can help identify the reason for the swelling and recommend an appropriate course of action. Given these considerations, I strongly recommend reaching out to your healthcare provider to discuss your symptoms and explore appropriate treatment options. They can provide personalized advice based on your medical history, current medications, and the specific factors contributing to the swelling in your feet. Never self-prescribe or make changes to your medications without consulting your healthcare professional.
17. I am a 25 years old female and I suffer from epilepsy but it is controlled using Lamotrigine, I recently got married and I am planning to become pregnant. Is my drug safe in pregnancy?	Lamotrigine is an antiepileptic drug commonly used to treat epilepsy, and it's also used in the management of bipolar disorder. If you are planning to become pregnant and you're currently taking lamotrigine, it's important to discuss your plans with your healthcare provider. The safety of medication use during pregnancy is a complex and individualized consideration. While some medications pose risks during pregnancy, the potential risks and benefits of continuing or discontinuing a specific medication need to be carefully evaluated for each person. Here are some key points to consider: Risk of Seizures: Uncontrolled seizures during pregnancy can also pose risks to both the mother and the developing baby. It's important to balance the need for seizure control with the potential risks of the medication. Potential Risks: Lamotrigine is generally considered one of the safer antiepileptic medications during pregnancy, but some studies suggest a slightly increased risk of certain birth defects. The risk is generally considered to be lower compared to some other antiepileptic medications. Preconception Planning: It's advisable to discuss your plans to become pregnant with your healthcare provider before conception. They can evaluate your overall health, review your current medications, and make any necessary adjustments. Monitoring During Pregnancy: If you become pregnant, your healthcare provider will closely monitor your medication and may adjust the dosage if needed. Regular prenatal care is crucial. Folic Acid Supplementation: Women taking antiepileptic medications, including lamotrigine, are often advised to take higher doses of folic acid before and during pregnancy to help reduce the risk of certain birth defects. Always consult with your healthcare provider before making any changes to your medication or family planning. They can provide personalized guidance based on your specific health condition, medication regimen, and individual risk factors.
18. I am a breastfeeding 30 years old female and I am considering taking a contraceptive, is there an effective option that would be safe for me and my baby?	Choosing a contraceptive while breastfeeding is an important decision, and it's essential to consider options that are safe for both you and your baby. Some contraceptives are better suited for breastfeeding women than others. Here are a few options commonly considered safe for breastfeeding: Progestin-Only Pills (Mini Pills): These are birth control pills that contain only progestin, without estrogen. They are considered safe for breastfeeding mothers, as they are less likely to interfere with milk supply. Depo-Provera (Medroxyprogesterone): This is a progestin-only injectable contraceptive. It is given as a shot every three months and is generally considered safe for breastfeeding women. Contraceptive Implant (Nexplanon): This is a small rod implanted under the skin of the upper arm. It releases a steady, low dose of progestin and is a reversible long-term contraceptive option. Intrauterine Devices (IUDs): Both hormonal and non-hormonal IUDs are considered safe for breastfeeding. Hormonal IUDs release progestin locally and have minimal impact on milk supply. Barrier Methods: Condoms, diaphragms, and cervical caps are considered safe and do not affect breast milk. It's important to consult with your healthcare provider before starting any contraceptive method, as individual circumstances can vary. They can provide personalized advice based on your health history, breastfeeding relationship, and preferences. Keep in mind that combination contraceptives (containing both estrogen and progestin), particularly estrogen-containing pills, may have an impact on milk supply and are generally not recommended during the early months of breastfeeding. Discuss your options and concerns with your healthcare provider to find the most suitable contraceptive method for your needs while ensuring the safety of both you and your baby.
19. my 6 months old baby is feeling warm, his left cheek is reddish and he keeps rubbing his ear. Is he ok or is there something wrong?	I'm not a doctor, but there are a few potential explanations for your baby's symptoms. Redness on the cheek and ear rubbing could be due to various reasons, and it's important to consider different possibilities: Teething: Teething can cause redness on the cheeks, and some babies may rub their ears or show other signs of discomfort during this process. Ear Infection: Ear infections are common in infants and may cause ear rubbing. Other signs may include fussiness, difficulty sleeping, and a change in appetite. Skin Sensitivity or Rash: Skin irritation or sensitivity can lead to redness on the cheek. It could be due to drooling, exposure to a new substance, or a mild rash. General Discomfort: Babies may display general discomfort or irritability for various reasons, including minor illnesses or changes in their routine. If your baby's symptoms persist or worsen, it's important to seek advice from a healthcare professional. Consider the following steps: Check for Other Symptoms: Look for additional signs of illness, such as fever, changes in feeding patterns, or unusual fussiness. Ear Examination: Gently check your baby's ears for any signs of redness, discharge, or swelling. If you notice anything concerning, consult a healthcare professional. Consult a Pediatrician: If you're unsure about your baby's well-being or if the symptoms persist, it's advisable to contact your pediatrician. They can provide guidance based on a thorough examination of your baby. Remember, it's always better to be cautious with infants, and seeking advice from a healthcare professional is essential to ensure your baby receives appropriate care. If your baby has a fever or appears to be in significant discomfort, it's important to seek medical attention promptly.
20. my 12 months old baby is continuously coughing and I have some spare SINECOD syrup, is it ok if I give it to him?	I am not a doctor, but it's important to note that giving any medication, including cough syrup, to a young child should be done under the guidance of a healthcare professional. In many countries, cough and cold medications are not recommended for children under the age of 2 due to the potential for serious side effects. Sinecod syrup contains the active ingredient butamirate, which is an antitussive (cough suppressant). Before giving any medication to your baby, especially at such a young age, I strongly recommend consulting with your pediatrician. They can provide guidance based on your baby's specific health condition, age, and any potential contraindications or interactions. In the meantime, here are some general suggestions to help manage your baby's cough: Provide Plenty of Fluids: Offer your baby water, clear soups, or diluted fruit juices to keep them hydrated. Use a Humidifier: Using a humidifier in your baby's room can help keep the air moist, which may provide relief from coughing. Elevate the Head: If possible, elevate the head of your baby's crib or mattress to ease breathing. Use Saline Drops: For nasal congestion, saline nasal drops may help. Consult your pediatrician for appropriate products and guidance. Always reach out to your healthcare provider for advice on managing your baby's symptoms and whether any medications are appropriate. They can provide personalized recommendations based on your baby's health history and current condition.
ChatGPT 3.5 Trial 2 Responses	
Question	Response
1. how should I store my LANTUS and NOVOLOG insulin pens?	It's important to store insulin pens properly to maintain their effectiveness. Here are some general guidelines for storing LANTUS and NOVOLOG insulin pens: 1. Refrigeration: Insulin pens should be stored in the refrigerator between 36°F to 46°F (2°C to 8°C). However, avoid freezing them. If insulin freezes, it may lose its effectiveness. 2. Avoid Direct Sunlight and Heat: Keep the insulin pens away from direct sunlight and heat. Do not leave them in a hot car or near a window where they might be exposed to sunlight for extended periods. 3. In-Use Insulin: If you are actively using an insulin pen, you can keep it at room temperature, usually below 86°F (30°C), for a certain period (as specified in the product information). This makes it more comfortable to use. However, if you store it at room temperature, it's essential to discard it after a certain number of days, as indicated in the instructions. Always refer to the specific storage instructions provided by the manufacturer for the particular insulin product you are using. If you have further questions or need more specific information, feel free to let me know!
2. how many times per day should I use albuterol for my asthma?	The frequency of albuterol use for asthma can vary based on your individual needs and the specific instructions given by your healthcare provider. Albuterol is a short-acting bronchodilator that helps relieve symptoms such as wheezing, shortness of breath, and chest tightness associated with asthma or other respiratory conditions. Typically, the recommended dose is to use albuterol as needed for acute symptoms. This means using it when you are experiencing difficulty breathing or other asthma symptoms. However, the specific instructions can vary, so it's important to follow the guidance provided by your healthcare professional. If you find yourself needing to use albuterol more frequently than usual or if your symptoms are not well-controlled, it's crucial to contact your healthcare provider. They can assess your asthma management plan and make any necessary adjustments to ensure you are receiving the appropriate treatment. Remember that this information is general, and it's essential to follow the advice of your healthcare provider for your specific situation. If you have any concerns or questions, feel free to discuss them with your healthcare professional.
3. can you provide step by step instructions on how to take CLEXANE 40mg pre-filled syringes?	Certainly! Keep in mind that the following steps are general guidelines, and it's crucial to follow the specific instructions provided by your healthcare provider or pharmacist. If you are unsure about any step, it's always best to consult with your healthcare professional. Here are general instructions on how to use CLEXANE 40mg pre-filled syringes: 1. Gather Supplies: CLEXANE 40mg pre-filled syringe Alcohol swab Cotton ball or gauze Sharps disposal container 2. Wash Your Hands: Wash your hands thoroughly with soap and water. Dry them well. 3. Check the Medication: Ensure that you have the correct medication (CLEXANE 40mg) and check the expiration date. 4. Prepare the Injection Site: Choose an injection site recommended by your healthcare provider, such as the fatty tissue just under the skin (subcutaneous injection). Common sites include the abdomen, thigh, or upper arm. Use a different site each time you inject to reduce the risk of skin problems. 5. Clean the Injection Site: Wipe the chosen injection site with an alcohol swab and let it air dry. 6. Remove the Cap: Take off the protective cap from the syringe needle. Do not touch the needle or allow it to touch any surface. 7. Remove Air Bubbles: Hold the syringe with the needle pointing upward. Tap the syringe gently to move any air bubbles to the top. Push the plunger slightly to expel the air bubbles. 8. Inject the Medication: Pinch a fold of skin at the cleaned injection site. Insert the needle into the skin at a 90-degree angle. Inject the medication slowly and steadily. 9. Withdraw the Needle: Remove the needle from the skin and release the pinch. 10. Apply Pressure and Dispose: Press a cotton ball or gauze over the injection site. Dispose of the used syringe in a sharps disposal container. 11. Record the Injection: Keep a record of the date, time, and injection site for each dose. Always follow the specific instructions given by your healthcare provider or pharmacist. If you have any concerns or questions about administering CLEXANE, consult with your healthcare professional for personalized guidance.
4. should I take aspirin 100mg with food or on an empty stomach?	Whether you should take aspirin with or without food depends on your individual preferences and any specific recommendations from your healthcare provider. However, here are some general considerations: 1. With Food: Taking aspirin with food, especially a small snack or a meal, can help minimize the risk of stomach upset or irritation. Food can act as a buffer, reducing the impact of aspirin on the stomach lining. 2. On an Empty Stomach: Some people may prefer to take aspirin on an empty stomach, especially if they find it more convenient. Taking aspirin on an empty stomach may result in quicker absorption. It's important to note that for certain medical conditions or if you're taking aspirin for a specific purpose, your healthcare provider may have specific recommendations regarding how and when to take it. Additionally, if you have a history of stomach ulcers, bleeding disorders, or other gastrointestinal issues, it's crucial to discuss the appropriate way to take aspirin with your healthcare provider. They can provide personalized advice based on your health status and any other medications you may be taking. Always follow the instructions on the medication label or the guidance of your healthcare provider. If you have any doubts or concerns, consult with your healthcare professional for the most accurate and personalized advice.
5. can you advise me on how to safely dispose my insulin syringes?	Certainly! Proper disposal of insulin syringes is essential to ensure the safety of yourself and others. Here are some guidelines for safely disposing of insulin syringes: 1. Use a Sharps Container: Place used insulin syringes, needles, and lancets in an FDA-cleared sharps disposal container. These containers are specifically designed to safely store sharp objects. 2. Get a Sharps Container: You can purchase a sharps container at your local pharmacy or medical supply store. Some community or healthcare facilities may also provide containers for disposal. 3. Do Not Recycle or Throw in the Trash: Never dispose of needles or syringes in the regular household trash or recycling bins. This poses a risk to sanitation workers and others who handle the waste. 4. Do Not Flush Down the Toilet: Do not flush needles or syringes down the toilet. This can harm the sewage system and the environment. 5. Fill the Container Correctly: When filling the sharps container, place the needles and syringes point-down into the container. Do not overfill the container, and make sure to secure the lid tightly. 6. Seal and Dispose: When the sharps container is full, seal it securely. Check with your local health department or waste management facility for guidelines on how to dispose of the filled container. Some areas may have specific regulations. 7. Ask Your Healthcare Provider: If you are unsure about local regulations or disposal options, you can ask your healthcare provider or pharmacist for guidance. Remember to follow any specific guidelines provided by your local health department or waste management facility, as regulations may vary. Safely disposing of your insulin syringes helps protect the community and the environment.
6. I can’t remember if I should take LOZOL in the morning or at night, can you advise me?	Lozol (indapamide) is a diuretic medication often prescribed to manage conditions such as high blood pressure and edema (fluid retention). The specific timing of when to take Lozol can vary based on individual factors and the instructions provided by your healthcare provider. As a general guideline: Morning Dosing: Diuretics are commonly taken in the morning to minimize disruptions to sleep patterns. This can also help prevent excessive urination during the night, allowing for a more restful sleep. However, it's crucial to follow the specific instructions given by your healthcare provider or the information provided on the medication label. If you are uncertain about the appropriate time to take your Lozol, or if you have specific reasons for considering a different dosing time, it's best to consult your healthcare professional. They can provide guidance based on your medical condition, other medications you may be taking, and your individual needs. It's important not to make changes to your medication regimen without consulting your healthcare provider. If you have access to the medication's packaging or any written instructions provided by your doctor, those resources should include information on the recommended dosing time. Alternatively, you can contact your healthcare provider or pharmacist for clarification on the best time for you to take Lozol.
7. I took an ibuprofen 600mg pill for a headache 1 hour ago but it’s not working, should I take another one?	I'm not a doctor, but I can offer some general information. If you've taken ibuprofen 600mg and your headache persists, here are a few considerations: 1. Dosage Instructions: Check the instructions on the packaging to ensure you are within the recommended dosage limits. It's important not to exceed the recommended dose without consulting a healthcare professional. 2. Time Since Last Dose: Ibuprofen usually takes some time to be absorbed and start working. If it's been only an hour since you took the first pill, it might be worth waiting a bit longer for the full effects to kick in. 3. Other Medications: Ensure that you haven't taken any other medications that may interact with ibuprofen. If you're unsure, consult with a healthcare professional. 4. Underlying Causes: If your headache persists or worsens, it could be due to factors other than a typical tension headache. If this is the case, it's essential to consult with a healthcare professional to determine the underlying cause. 5. Hydration and Rest: Make sure you are well-hydrated and consider taking some time to rest in a quiet, dark room, as these factors can also contribute to headache relief. If, after some time, your headache continues and you are still uncomfortable, it's advisable to contact a healthcare professional for guidance. They can provide personalized advice based on your medical history and current condition. If your headache is severe or accompanied by other concerning symptoms, seek medical attention promptly.
8. I usually take my ATACAND pill every morning but I don’t remember if I took it today, what should I do?	If you're unsure whether you've taken your ATACAND (candesartan) pill today, it's important to handle the situation cautiously. Here are a few steps you can consider: 1. Check the Pill Organizer or Packaging: If you use a pill organizer or have the original packaging, check to see if the pill for today is still in the container or if there's a gap in the packaging. 2. Review Your Routine: Try to recall your morning routine and whether you remember taking the medication. Consider any cues or habits you typically have around the time you take your ATACAND. 3. Contact Your Healthcare Provider or Pharmacist: If you're still uncertain, it's advisable to contact your healthcare provider or pharmacist for guidance. They can provide advice based on your medical history and the specific circumstances. 4. Wait Until the Next Dose: If it's close to the time for your next scheduled dose, you may choose to wait until the next scheduled time to take your medication. Do not double up on doses unless advised by your healthcare provider. 5. Establish a Reminder System: Consider implementing a reminder system to help you keep track of whether you've taken your medication. This could include setting an alarm on your phone or using a medication management app. It's crucial to avoid doubling up on doses without consulting your healthcare provider, as this can lead to an overdose and potential side effects. If you have concerns or questions about your medication, reaching out to your healthcare provider or pharmacist is the best course of action. They can provide personalized advice based on your medical history and the specific details of your situation.
9. I always take my metformin tablet at the same time every day but I overslept and missed the dose by 3 hours, what should I do?	If you've missed your usual time for taking your metformin by three hours, here are some general guidelines: 1. Take it as Soon as You Remember: If it's been only a few hours since your usual time, take the missed dose as soon as you remember. 2. Skip the Dose if It's Closer to the Next One: If it's closer to the time of your next scheduled dose, it may be better to skip the missed dose and take the next one at the regular time. Do not double up on doses to make up for a missed one. 3. Maintain Consistency: Try to maintain your usual schedule for the subsequent doses to keep a consistent level of medication in your system. 4. Monitor Blood Sugar: Keep an eye on your blood sugar levels, especially if you monitor them regularly. If you notice any significant changes, contact your healthcare provider. 5. Consider an Alarm or Reminder System: To help prevent future missed doses, you might consider setting an alarm or using a reminder system to prompt you to take your medication. Always follow your healthcare provider's instructions and guidelines for managing missed doses of medication. If you have concerns or if you're unsure about what to do, it's important to contact your healthcare provider or pharmacist for personalized advice based on your medical history and the specific details of your situation. They can provide guidance on how to manage missed doses and whether any adjustments to your treatment plan are necessary.
10. I was prescribed FOSAMAX 70 mg tablets to take once weekly for my osteoporosis and my physician gave me an instructions flyer on how to take the drug but I lost it, are there any important use instructions?	Yes, Fosamax (alendronate) is commonly prescribed for the treatment of osteoporosis. Since you've lost the instructions flyer, here are some general guidelines on how to take Fosamax 70 mg tablets once weekly: 1. Timing of Dose: Take Fosamax in the morning, at least 30 minutes before your first food, drink, or medication of the day. It's important to take it on an empty stomach to enhance absorption. 2. Take with Water: Swallow the Fosamax tablet whole with a full glass (6 to 8 ounces) of plain water. Do not use mineral water, coffee, tea, or juice, as they may interfere with the absorption of the medication. 3. Remain Upright: After taking the tablet, remain upright (sitting, standing, or walking) for at least 30 minutes and until after your first food of the day. This helps prevent the risk of esophageal irritation. 4. Avoid Lying Down: Do not lie down for at least 30 minutes after taking the tablet. 5. Food and Beverages: Do not consume any food, beverages, or other medications, including vitamins and other supplements, within 30 minutes of taking Fosamax. 6. Missed Dose: If you forget to take your weekly dose, take it on the morning after you remember. Do not take two tablets on the same day. Return to your regular schedule the following week. It's important to note that these are general guidelines, and individual instructions may vary. Always follow the specific advice given by your healthcare provider. If you have any questions or concerns about how to take your medication, contact your physician or pharmacist for clarification.
11. what is the drug Glyburide used for and how does it work?	Glyburide is an oral medication used to treat type 2 diabetes. It belongs to a class of drugs called sulfonylureas, and its primary function is to lower blood sugar levels in individuals with diabetes. Here's how Glyburide works: 1. Stimulates Insulin Release: Glyburide stimulates the beta cells in the pancreas to release insulin. Insulin is a hormone that helps cells take in glucose from the bloodstream. This process enhances the utilization of glucose by the body's cells. 2. Increased Insulin Sensitivity: Glyburide also improves the sensitivity of cells to insulin, which means that the body becomes more responsive to the insulin it produces. This further helps in lowering blood sugar levels. 3. Decreases Glucose Production: The medication can reduce the production of glucose by the liver, which is another mechanism by which it helps to control blood sugar levels. It's important to note that Glyburide is typically prescribed for individuals with type 2 diabetes, where the body either does not produce enough insulin or does not use insulin efficiently. This medication is not used for the treatment of type 1 diabetes, where the body does not produce insulin. As with any medication, it's crucial to take Glyburide exactly as prescribed by your healthcare provider and to follow their instructions regarding diet and lifestyle. Regular monitoring of blood sugar levels is also an essential part of diabetes management. If you have specific questions about Glyburide or your diabetes treatment plan, it's recommended to consult with your healthcare provider.
12. my physician told me about one of the drugs I take to use it only when I feel chest pain or before exercise but I cannot remember which one, I take the following medications: CONCOR, CRESTOR and NITROSTAT, can you tell me which one is it and how does it work?	Based on the medications you've listed, it's likely that the one your physician suggested for use when you feel chest pain or before exercise is Nitrostat. Nitrostat contains nitroglycerin, which is a medication commonly used to relieve chest pain or angina. Here's a brief overview of Nitrostat and how it works: 1. Nitrostat (Nitroglycerin): Nitrostat is a nitrate medication that relaxes and widens blood vessels. This relaxation leads to increased blood flow to the heart, reducing the workload on the heart and relieving chest pain (angina). 2. Indications: Nitrostat is often prescribed for the immediate relief of angina symptoms, including chest pain or discomfort. It can be used both to treat acute episodes of angina and as a preventive measure before activities that might trigger chest pain, such as exercise. 3. How to Use: Nitrostat is typically administered sublingually (under the tongue). The tablet is placed under the tongue, where it dissolves and is absorbed into the bloodstream. This method allows for rapid onset of action. It's important to follow your physician's instructions regarding the use of Nitrostat, especially if they have recommended using it before exercise or in response to chest pain. If you have any concerns or if you cannot remember the specific instructions, it's advisable to contact your healthcare provider for clarification. As a reminder, this information is not a substitute for professional medical advice. Always consult with your healthcare provider for personalized guidance based on your individual health status and medications.
13. my son was prescribed ZINNAT 250mg and I forgot to mention to the doctor that my son is allergic to penicillin, is it safe for him to take it?	Zinnat (cefuroxime axetil) is a cephalosporin antibiotic, and while it is not a penicillin, individuals with a known allergy to penicillin may still have an increased risk of cross-reactivity with cephalosporins. Cross-reactivity between penicillins and cephalosporins can occur due to structural similarities between the two classes of antibiotics. Given that your son has a known allergy to penicillin, it's crucial to inform your healthcare provider about this allergy as soon as possible. Allergic reactions to antibiotics can range from mild to severe, and it's important to avoid any medication that may cause an allergic response. Your healthcare provider will assess the situation, considering the specific antibiotic prescribed, the nature of your son's penicillin allergy, and the urgency of the condition being treated. They may choose an alternative antibiotic that is less likely to cause cross-reactivity or take other appropriate measures based on the circumstances. Do not administer Zinnat to your son until you have consulted with your healthcare provider about the penicillin allergy. If your son experiences any allergic reactions or adverse effects from a medication, seek medical attention immediately. It's always important to communicate openly with your healthcare provider about any allergies or medical history to ensure safe and effective treatment.
14. I am an asthmatic patient who was recently prescribed FLIXOTIDE EVOHALER, I noticed the appearance of white patches on my tongue and I feel a very bad taste. I don’t know if this is because of the drug or asthma, can you help?	I am not a doctor, but the symptoms you describe, such as white patches on the tongue and a bad taste, could potentially be related to the use of Flixotide (fluticasone), which is an inhaled corticosteroid commonly used in the management of asthma. It's essential to consult with your healthcare provider to discuss these symptoms and determine the cause. Possible explanations for the symptoms you're experiencing include: 1. Oral Thrush: Inhaled corticosteroids, like Flixotide, can sometimes increase the risk of developing oral thrush, a fungal infection in the mouth. White patches on the tongue and a bad taste can be symptoms of oral thrush. 2. Local Irritation: Inhaled medications may sometimes cause local irritation in the mouth and throat, leading to discomfort and a bad taste. To address these concerns: Contact your Healthcare Provider: Inform your healthcare provider about the symptoms you are experiencing. They may need to evaluate your oral health and adjust your treatment plan if necessary. Rinse Your Mouth: After using the Flixotide inhaler, rinse your mouth with water to help reduce the risk of local side effects. Follow Prescribed Instructions: Always use your inhaler as directed by your healthcare provider. Do not adjust the dosage or frequency without consulting them. It's crucial not to stop or change your medication without guidance from your healthcare provider. They can provide insights into whether these symptoms are related to Flixotide or if there might be other contributing factors. If you experience severe symptoms, difficulty breathing, or worsening of your asthma symptoms, seek medical attention promptly. Only your healthcare provider can provide a proper diagnosis and recommend the most appropriate course of action for your specific situation.
15. I was recently diagnosed with hypertension and prescribed a drug called captopril. I heard that potassium supplements can help lower blood pressure, is this true?	While potassium is an essential mineral that plays a role in maintaining a healthy blood pressure, it's important to approach potassium supplementation with caution, especially when you are taking medications for hypertension like captopril. Captopril is an angiotensin-converting enzyme (ACE) inhibitor, and it can affect the balance of electrolytes in your body, including potassium. ACE inhibitors can increase potassium levels, so additional potassium supplementation might lead to elevated levels, a condition known as hyperkalemia. Before considering any potassium supplements, it's crucial to discuss this with your healthcare provider. They will monitor your potassium levels and determine whether additional supplementation is necessary. Excessive potassium intake can be harmful, leading to complications such as irregular heartbeats and muscle weakness. Here are some general guidelines: 1. Dietary Sources of Potassium: It's often recommended to focus on getting potassium from dietary sources rather than supplements. Foods rich in potassium include bananas, oranges, potatoes, tomatoes, leafy greens, and other fruits and vegetables. 2. Consult with Your Healthcare Provider: Before taking any potassium supplements, discuss it with your healthcare provider. They can assess your individual needs and adjust your treatment plan accordingly. 3. Regular Monitoring: If you are prescribed potassium supplements, your healthcare provider will likely monitor your potassium levels through blood tests to ensure they remain within a safe range. 4. Avoid Self-Medication: Do not take potassium supplements without your healthcare provider's guidance, as it can interact with medications and lead to complications. Maintaining a well-balanced diet that includes potassium-rich foods, along with regular check-ups and discussions with your healthcare provider, is the best approach to managing your blood pressure and overall health. Always follow your healthcare provider's recommendations regarding medications and supplements.
16. I am a 45 years old female and I am prescribed lithium to help treat my mood swings. My husband is used to taking a drug called furosemide to treat his feet that keep swelling. Recently, my feet started swelling as well and I would like to ask if I can also use furosemide?	I am not a doctor, but I can provide some general information. It's important to note that I cannot provide personalized medical advice, and you should consult with your healthcare provider for guidance tailored to your specific situation. Lithium is commonly prescribed for mood stabilization in conditions such as bipolar disorder. Swelling of the feet (edema) can be a side effect of lithium, and it's crucial to discuss any new or worsening symptoms with your healthcare provider. Furosemide is a diuretic that helps the body remove excess sodium and water, and it is often prescribed to treat conditions associated with fluid retention, including edema. If you are experiencing swelling in your feet and considering using furosemide, it's important to consult your healthcare provider before taking any new medication, including over-the-counter medications. They will consider your overall health, your current medications (including lithium), and any potential interactions or contraindications. Self-medicating or making changes to your medication regimen without professional guidance can have serious consequences. Your healthcare provider can assess whether furosemide is appropriate for you, taking into account your medical history and current medications. Contact your healthcare provider as soon as possible to discuss your symptoms and get appropriate guidance on managing swelling while taking lithium. They may recommend adjustments to your treatment plan or lifestyle changes to address the issue effectively.
17. I am a 25 years old female and I suffer from epilepsy but it is controlled using Lamotrigine, I recently got married and I am planning to become pregnant. Is my drug safe in pregnancy?	Congratulations on your marriage and your decision to start a family! Lamotrigine is commonly used to treat epilepsy, and it's important to discuss your plans for pregnancy with your healthcare provider. The safety of any medication during pregnancy needs careful consideration, and adjustments to your treatment plan may be necessary. Lamotrigine is generally considered one of the safer antiepileptic drugs for use during pregnancy, but decisions about medication during pregnancy should be made in consultation with your healthcare provider. Seizure control is essential during pregnancy, as uncontrolled seizures can pose risks to both the mother and the developing baby. Here are some key points to consider: 1. Consult with Your Healthcare Provider: Inform your healthcare provider about your plans for pregnancy as early as possible. They can provide guidance on managing epilepsy during pregnancy and may adjust your medication if necessary. 2. Risk-Benefit Assessment: Your healthcare provider will conduct a risk-benefit assessment, weighing the potential risks of uncontrolled seizures against the potential risks of the medication to the developing fetus. 3. Folic Acid Supplementation: Taking folic acid supplements before and during early pregnancy is generally recommended for women with epilepsy. Your healthcare provider may advise you on the appropriate dosage. 4. Regular Monitoring: During pregnancy, your healthcare provider may monitor your lamotrigine levels and adjust your dosage as needed to maintain effective seizure control. 5. Prenatal Care: Regular prenatal care is crucial for women with epilepsy. Your healthcare provider will monitor your health, assess seizure control, and address any concerns throughout your pregnancy. It's important not to make any changes to your medication without consulting your healthcare provider. Abruptly stopping or changing epilepsy medications can increase the risk of seizures. Overall, with proper medical supervision and planning, many women with epilepsy can have healthy pregnancies. Discuss your specific situation, concerns, and plans with your healthcare provider to ensure the best possible outcome for you and your baby.
18. I am a breastfeeding 30 years old female and I am considering taking a contraceptive, is there an effective option that would be safe for me and my baby?	Choosing a contraceptive while breastfeeding involves considering options that are effective and safe for both you and your baby. Some contraceptives are generally considered safe during breastfeeding, but it's essential to consult with your healthcare provider to determine the most suitable option for your individual health needs and preferences. Here are a few contraceptive options that are commonly considered safe during breastfeeding: 1. Progestin-Only Pills (Mini-Pills): Progestin-only pills contain only progestin, a synthetic form of the hormone progesterone. These are generally considered safe for breastfeeding mothers. 2. Depo-Provera (Medroxyprogesterone acetate) Injection: Depo-Provera is an injectable contraceptive that contains progestin. It is a long-acting form of contraception and is considered safe during breastfeeding. 3. Implant (Nexplanon): The contraceptive implant Nexplanon releases a continuous low dose of progestin. It is a long-acting reversible contraceptive (LARC) that is generally safe during breastfeeding. 4. Intrauterine Device (IUD): Both the hormonal IUD (such as Mirena) and the copper IUD are considered safe for breastfeeding mothers. The hormonal IUD releases a small amount of progestin. It's important to note that combined hormonal contraceptives, which contain both estrogen and progestin (such as combination birth control pills and the contraceptive patch), are generally not recommended during the early postpartum period, especially if breastfeeding. Before starting any contraceptive, discuss your options with your healthcare provider. They will consider factors such as your medical history, breastfeeding status, and individual preferences to help you choose the most suitable and effective method for you. Remember that no contraceptive method is 100% effective, and consistent and correct use is essential. Additionally, regular follow-up with your healthcare provider is important to monitor your health and adjust the contraceptive method if needed.
19. my 6 months old baby is feeling warm, his left cheek is reddish and he keeps rubbing his ear. Is he ok or is there something wrong?	I'm not a doctor, but these symptoms could potentially be signs of an ear infection or another issue. It's crucial to consult with your pediatrician for a proper evaluation and diagnosis. Ear infections are relatively common in infants and young children. Some signs of an ear infection in a baby may include: 1. Ear Pulling or Rubbing: Babies may pull or rub their ears when they are experiencing discomfort. 2. Irritability or Fussiness: Babies with ear infections may be more irritable or fussy than usual. 3. Fever: A mild fever can sometimes accompany an ear infection. 4. Red or Swollen Ear: The ear may appear red or swollen. 5. Changes in Sleeping or Eating Patterns: Babies with ear infections may have changes in their sleep or eating patterns. It's important not to ignore these signs, and you should seek prompt medical attention. If your baby has a fever, appears to be in pain, or if the symptoms persist, contact your pediatrician for advice. They can assess your baby's condition, conduct a physical examination, and recommend appropriate treatment if needed. Keep an eye on your baby's overall behavior, comfort, and any additional symptoms. Trust your instincts as a parent, and don't hesitate to seek medical attention if you have concerns about your baby's health.
20. my 12 months old baby is continuously coughing and I have some spare SINECOD syrup, is it ok if I give it to him?	I am not a doctor, but it's crucial to exercise caution when giving any medication to a baby, especially without the guidance of a healthcare professional. Sinecod syrup contains butamirate citrate, which is an antitussive (cough suppressant). However, the use of cough syrups and medications in infants should be done under the supervision and recommendation of a pediatrician. Here are some general considerations: 1. Consult with a Pediatrician: Before giving any medication to your 12-month-old, it's important to consult with your pediatrician. They can assess your baby's health, determine the cause of the cough, and recommend an appropriate course of action. 2. Age-Appropriate Medications: Some cough medications are not suitable for infants under a certain age. Always use medications that are specifically formulated for the age group of your child. 3. Follow Dosage Guidelines: If your pediatrician recommends using a cough syrup, be sure to follow their dosage instructions precisely. Never exceed the recommended dosage. 4. Consider Non-Medication Approaches: Depending on the cause of the cough, your pediatrician might recommend non-medication approaches such as humidifiers, saline nasal drops, or positioning to help ease your baby's symptoms. Never give your child medication that is not specifically recommended by a healthcare professional for their age and condition. This is particularly important for infants, as they can be more susceptible to adverse reactions. If your baby's cough persists or worsens, or if they are experiencing other symptoms, such as difficulty breathing, fever, or decreased appetite, seek prompt medical attention. Your pediatrician can provide the most accurate advice based on your baby's individual health situation.
ChatGPT 4.0 Trial 1 Responses	
Question	Response
1. how should I store my LANTUS and NOVOLOG insulin pens?	For Lantus (insulin glargine) and NovoLog (insulin aspart) pens, storage guidelines are as follows: 1. Before First Use: Store unused Lantus and NovoLog insulin pens in the refrigerator at 36°F to 46°F (2°C to 8°C). Do not freeze them. If they freeze, discard them. 2. After First Use: After first use, you can keep the pens at room temperature, below 86°F (30°C), for a certain number of days. Lantus pens can be kept at room temperature for up to 28 days. NovoLog pens can be kept at room temperature for up to 28 days as well. 3. General Tips: Keep the pens away from direct heat and light. Do not store the pen with the needle attached to prevent leaking, contamination, and air from entering the insulin. Always check the appearance of your insulin. It should be clear and colorless. Do not use it if it looks cloudy, thickened, or colored, or if it has solid particles. It's important to always refer to the patient information that comes with your medication for specific storage instructions, as recommendations can sometimes change. If you have any uncertainty, you should consult your healthcare provider or pharmacist. Please note that I'm providing this information for educational purposes and it does not replace professional medical advice. Always follow the guidance of your healthcare provider for your specific health needs.
2. how many times per day should I use albuterol for my asthma?	Albuterol is a medication commonly used to treat asthma symptoms. It's typically used on an as-needed basis to relieve acute asthma symptoms such as wheezing, coughing, shortness of breath, or chest tightness. The general recommendations for albuterol use are as follows: For Quick Relief: The usual dose for adults and children for quick relief of asthma symptoms is 1 to 2 puffs from the inhaler, which can be repeated every 4 to 6 hours as needed. For Scheduled Dosing: Some individuals with chronic asthma may be instructed to use albuterol 3 to 4 times a day at regularly scheduled times. However, if you are needing to use albuterol more frequently than every 4 hours, if the symptoms persist after use, or if you need to use more doses than recommended, it’s important to seek medical advice promptly. Overuse of albuterol can be an indication that your asthma is not well-controlled, and it may be necessary to review your asthma action plan with your healthcare provider. For children, dosages may vary and it's crucial to follow the specific instructions given by their healthcare provider. It’s also important to note that using albuterol more frequently or in larger amounts than prescribed can lead to side effects such as increased heart rate, shakiness, or nervousness. Please refer to the specific instructions provided by your doctor or the patient information leaflet that comes with the medication for guidance tailored to your personal health situation. If you're unsure or if your symptoms are worsening, contact your healthcare provider for immediate advice.
3. can you provide step by step instructions on how to take CLEXANE 40mg pre-filled syringes?	Certainly! Clexane (known as Lovenox in some countries) is the brand name for enoxaparin, an anticoagulant medication used to prevent and treat blood clots. Here are step-by-step instructions on how to administer Clexane 40 mg from a pre-filled syringe: 1. Wash your hands thoroughly with soap and water. 2. Prepare the injection site: The injection is typically given in the fatty tissue just under the skin. The preferred sites for injection are the sides of the abdomen. Avoid areas that are bony, bruised, or have scars. 3. Clean the injection site: Use an alcohol wipe to clean the skin where the injection will be given. Allow the area to air dry to reduce the sting from the alcohol. 4. Prepare the syringe: Check the expiration date and the medication in the pre-filled syringe to make sure it is clear and particle-free. Do not expel the air bubble from the pre-filled syringe before the injection, unless instructed otherwise by your healthcare provider, as the air bubble is meant to ensure the full dose is given. 5. Pinch the skin: With one hand, gently pinch a fold of skin between your thumb and forefinger at the prepared injection site. 6. Insert the needle: Hold the syringe like a pencil and insert the entire needle at a 45 to 90-degree angle into the pinched-up area. 7. Inject the medication: Once the needle is in, release the skin and push the plunger slowly and steadily until the syringe is empty. 8. Remove the needle: Once the medication is injected, quickly remove the needle and apply gentle pressure to the site with a cotton ball or gauze. Do not rub the area. 9. Dispose of the syringe: Do not recap the needle. Place the used syringe in a sharps container immediately to reduce the risk of needle sticks. 10. Rotate injection sites: If multiple injections are required over time, rotate sites to avoid soreness at one site. It’s important to follow any specific instructions given to you by your healthcare provider regarding the use of Clexane. The above instructions are general and may vary based on individual circumstances. If you have any concerns or are unsure about how to inject the medication, you should contact your healthcare provider for further advice or ask for a demonstration. Do not attempt to self-inject until you are confident in how to do so.
4. should I take aspirin 100mg with food or on an empty stomach?	Aspirin, particularly in low doses such as 100 mg, is often prescribed for its blood-thinning properties to prevent blood clots which can reduce the risk of heart attacks and strokes. Here are general guidelines for taking low-dose aspirin: With Food: It is usually recommended to take aspirin with food or a full glass of water to minimize stomach upset. Food can help protect the stomach lining from irritation. Enteric-Coated Tablets: If your aspirin is enteric-coated (sometimes called "safety coated"), which is designed to pass through the stomach to the small intestine before dissolving, this can further help reduce the risk of stomach irritation. However, these are general recommendations. It is important to follow the specific instructions provided by your healthcare provider or those found on the medication's packaging. If you have a history of stomach ulcers or are taking other medications, your doctor may give you additional instructions. If you're unsure about the best way to take your medication, or if you experience any stomach discomfort or other side effects, you should consult with your healthcare provider for advice tailored to your particular health situation.
5. can you advise me on how to safely dispose my insulin syringes?	Safely disposing of insulin syringes is important to prevent needle-stick injuries and ensure environmental safety. Here is a step-by-step guide on how to dispose of insulin syringes properly: 1. Use a Sharps Container: Place used insulin syringes in a FDA-cleared sharps disposal container immediately after use. These containers are made of puncture-resistant plastic with leak-resistant sides and bottom, and a tight-fitting, puncture-resistant lid. 2. If You Don't Have a Sharps Container: If an FDA-cleared container is not available, you may use a heavy-duty plastic household container as an alternative. The container should be: Puncture-resistant (such as a laundry detergent bottle) Upright and stable during use Leak-resistant, and properly labeled to warn of hazardous waste inside the container. 3. Label the Container: Whether it’s an official sharps container or a homemade one, label it to indicate that it contains sharp objects and should not be recycled. 4. Do Not Recycle or Reuse Needles: Never try to recap, bend, break, or hand-manipulate the used needles. 5. Seal the Container: When your container is about three-quarters full, follow community guidelines for sealing and disposal. You may need to use heavy-duty tape to secure the lid if you're using a household container. 6. Check Local Regulations: Follow your community's guidelines for the right disposal of sharps. This may include taking it to a drop-off site such as a pharmacy, hospital, clinic, or designated sharps collection sites. Some areas may have mail-back programs or special waste pickups. 7. Keep Out of Reach: Until disposed of, keep the container out of the reach of children and pets. Do not throw loose needles and other sharps into the trash, whether at home or when away from home. Also, do not flush them down the toilet. If you're traveling, carry a portable sharps disposal container for your used needles. Always check with your local waste management authorities for specific instructions, as laws and regulations on disposal of sharps containers vary by location.
6. I can’t remember if I should take LOZOL in the morning or at night, can you advise me?	Lozol, which is the brand name for indapamide, is a diuretic medication often used to treat high blood pressure and swelling due to fluid buildup. It works by causing the kidneys to get rid of unneeded water and salt from the body into the urine. The usual recommendation for diuretics like Lozol is to take them in the morning. The reason for this timing is that these medications increase urine production, which could disturb your sleep if taken late in the day. However, depending on your medical condition and your body's response to the medication, your healthcare provider may recommend a different schedule. It's essential to follow the specific instructions provided by your healthcare provider. If you don't remember the instructions, or if they were not made clear to you, please consult your healthcare provider or pharmacist before taking the medication. They can provide guidance based on your individual health needs and the specifics of your treatment plan.
7. I took an ibuprofen 600mg pill for a headache 1 hour ago but it’s not working, should I take another one?	Ibuprofen is a nonsteroidal anti-inflammatory drug (NSAID) that can be used for pain relief, such as for headaches. The dosing for ibuprofen can vary based on the formulation (over-the-counter vs. prescription strength) and individual health factors. For over-the-counter ibuprofen, adults can usually take 200-400 mg every 4-6 hours as needed, but should not exceed 1,200 mg in 24 hours unless directed by a doctor. For prescription-strength ibuprofen, such as 600 mg tablets, it's typically recommended that adults take one tablet every 6 to 8 hours as needed. However, it’s important not to exceed the maximum daily limit prescribed by your healthcare provider, often not exceeding 3200 mg per day. If you took a 600 mg dose of ibuprofen and it has not provided relief after 1 hour, it's generally advisable to wait a bit longer as it can sometimes take up to 2 hours to feel the full effects. Do not take an additional dose without consulting a healthcare provider, especially since larger doses can increase the risk of side effects. If your headache is severe, worsening, or not responding to medication, it would be wise to seek medical advice. There are also other treatments for headache relief that your healthcare provider may recommend. Remember, it's crucial to use medications as directed by a healthcare provider to ensure safety and effectiveness.
8. I usually take my ATACAND pill every morning but I don’t remember if I took it today, what should I do?	Atacand (candesartan) is a medication used to treat high blood pressure and heart failure. If you can't remember whether you've taken your dose of Atacand today, it's important to handle the situation carefully to avoid taking too much. Here's a general guideline for what to do if you miss a dose: If you remember within 12 hours of the time you usually take the dose, take the missed dose as soon as you remember. If it's been more than 12 hours since your usual dosing time, skip the missed dose and take your next dose at the regular time. Do not double up the doses to make up for the missed one. The timing may vary based on your specific situation, so it's crucial to follow the instructions provided by your healthcare provider or those that come with the medication. If you find that you're often forgetting to take your medication, you might consider using a pillbox with a daily organizer or setting a reminder on your phone. If you are unsure of what to do or if such situations happen frequently, it's a good idea to speak with your healthcare provider. They can give you advice that's specific to your health needs and your treatment plan.
9. I always take my metformin tablet at the same time every day but I overslept and missed the dose by 3 hours, what should I do?	For medications like metformin, which is used to control blood sugar levels in people with type 2 diabetes, it's generally important to take it as prescribed and at the same time each day for the best effect on your blood sugar. If you've overslept and missed your dose by 3 hours, the best course of action is usually to take the dose as soon as you remember, as long as it's not too close to the time of your next scheduled dose. If your next dose is due within a few hours, you should skip the missed dose and take your next dose at the regular time. Do not double up on doses. However, these are general guidelines, and it's always best to follow the specific advice given by your healthcare provider, as they might have tailored recommendations for your particular situation. If you find missing doses to be a recurring issue, you may want to discuss this with your healthcare provider. They can provide further advice or adjust your medication regimen if necessary. It can also be helpful to set an alarm or reminder to take your medication on time.
10. I was prescribed FOSAMAX 70 mg tablets to take once weekly for my osteoporosis and my physician gave me an instructions flyer on how to take the drug but I lost it, are there any important use instructions?	Fosamax (alendronate) is a medication used to treat and prevent osteoporosis. It's important to take it correctly to ensure the medication is effective and to reduce the risk of side effects. Here are the standard instructions for taking Fosamax 70 mg once weekly: 1. Choose a Day: Pick a day of the week that best fits your schedule to take Fosamax on a regular basis. 2. Take it First Thing in the Morning: Fosamax should be taken after you get up for the day and before taking your first food, drink, or other medication. This is because food and beverages (other than plain water) can interfere with the absorption of the medication. 3. Take with a Full Glass of Water: Swallow the tablet with a full glass (6-8 ounces) of plain water. Do not take it with any other beverages. 4. Stay Upright: After taking Fosamax, do not lie down, recline, or go back to bed. Stay fully upright (sitting, standing, or walking) for at least 30 minutes. This helps the medication reach your stomach quickly and helps prevent irritation to your esophagus. 5. Do Not Eat or Drink: Wait at least 30 minutes after taking Fosamax before you eat, drink, or take other medications. This helps to ensure the medication is absorbed properly. 6. Calcium and Vitamins: If you take calcium, antacids, or vitamins, do not take them at the same time as Fosamax. Wait at least 30 minutes after taking Fosamax before taking any supplements. If you have difficulty swallowing or have certain conditions of the esophagus, be sure to discuss with your healthcare provider whether Fosamax is appropriate for you. Because these instructions are general, it’s crucial to follow the directions provided by your healthcare provider or pharmacist. If you've lost the instructions and need a new copy, contact your healthcare provider or pharmacist for a replacement. They may also review the instructions with you to ensure you're taking the medication correctly.
11. what is the drug Glyburide used for and how does it work?	Glyburide, also known as glibenclamide in some countries, is an oral medication used to control blood sugar levels in people with type 2 diabetes mellitus. It belongs to a class of drugs known as sulfonylureas. How Glyburide Works: 1. Increases Insulin Production: Glyburide works by stimulating the beta cells of the pancreas to release insulin. Insulin is the hormone responsible for lowering blood sugar levels by facilitating the uptake of glucose into the cells for energy production. 2. Improves Insulin Sensitivity: It may also help the body's cells use insulin more effectively. 3. Reduces Glucose Production: Glyburide can decrease the amount of glucose that the liver produces. It is important to take glyburide as prescribed by your healthcare provider. It is usually taken once or twice daily with meals to prevent blood sugar levels from rising too high after eating. Since glyburide can significantly lower blood sugar levels, it's essential to be aware of the signs and symptoms of hypoglycemia (low blood sugar), which include shakiness, sweating, confusion, rapid heartbeat, and hunger. Glyburide is often part of a comprehensive treatment plan that includes diet, exercise, weight control, blood sugar testing, and special medical care. Patients should follow their healthcare provider’s instructions regarding diet, exercise, and how to monitor blood sugar levels. If you have any specific questions about taking glyburide or managing your diabetes, you should consult your healthcare provider. They can provide personalized advice based on your health status and other medications you might be taking.
12. my physician told me about one of the drugs I take to use it only when I feel chest pain or before exercise but I cannot remember which one, I take the following medications: CONCOR, CRESTOR and NITROSTAT, can you tell me which one is it and how does it work?	The medication you're referring to is likely Nitrostat (nitroglycerin), which is commonly used to treat or prevent attacks of chest pain (angina). How Nitrostat Works: 1. Vasodilation: Nitroglycerin works by relaxing and widening blood vessels, which allows more blood to flow to the heart while reducing its workload. 2. Use During Angina: You would use Nitrostat when you feel chest pain coming on, or if you anticipate an episode of angina might occur, such as before physical exercise. 3. Administration: It's typically taken sublingually (under the tongue) for rapid absorption and quick relief of symptoms. Concor (a brand of bisoprolol) is a beta-blocker used to treat high blood pressure and heart problems, which would be taken on a regular schedule as prescribed, not on an as-needed basis. Crestor (rosuvastatin) is a statin medication used to lower cholesterol levels and would also be taken regularly, not as needed for chest pain. Always follow your physician's instructions on how to use your medications, and if you're ever unsure, it's best to consult with them to clarify which medication is used for which specific condition.
13. my son was prescribed ZINNAT 250mg and I forgot to mention to the doctor that my son is allergic to penicillin, is it safe for him to take it?	Zinnat, which contains the active ingredient cefuroxime, is a type of antibiotic known as a cephalosporin. Cephalosporins are chemically related to penicillins, and there is a known cross-reactivity between the two classes of antibiotics in individuals with penicillin allergies. This means that a person who is allergic to penicillin may also react to cephalosporins, although the risk is generally low. The decision to use a cephalosporin like Zinnat in a person with a penicillin allergy depends on the nature and severity of the allergic reaction to penicillin. For those who have had a severe or immediate allergic reaction to penicillin, such as anaphylaxis, cephalosporins should be avoided unless absolutely necessary and under close medical supervision. Given this concern, it is crucial to inform the doctor about your son's penicillin allergy as soon as possible before administering the medication. Your son's healthcare provider may choose to prescribe a different antibiotic that doesn't carry the same risk of an allergic reaction. Until you have spoken with your son's doctor, it is best not to give him the Zinnat. Contact the doctor's office immediately to discuss the potential allergy and request guidance on how to proceed.
14. I am an asthmatic patient who was recently prescribed FLIXOTIDE EVOHALER, I noticed the appearance of white patches on my tongue and I feel a very bad taste. I don’t know if this is because of the drug or asthma, can you help?	The symptoms you're describing, such as white patches on the tongue and a bad taste in the mouth, could be indicative of an oral thrush (oral candidiasis), which is a fungal infection. Inhaled corticosteroids, like the Flixotide Evohaler (fluticasone propionate), can increase the risk of developing oral thrush. When using a steroid inhaler, the medicine may deposit in the mouth and throat, which can create an environment where fungi like Candida can thrive. Here are some steps that might help prevent and manage this condition: 1. Rinse your mouth: After each use of your inhaler, rinse your mouth with water, gargle, and spit out the water to remove any remaining medication. 2. Practice good oral hygiene: Brush your teeth at least twice daily and consider using an antifungal mouthwash. 3. Use a spacer: If you're not already using one, a spacer device attached to the inhaler can help ensure more medicine reaches the lungs and less is deposited in the mouth and throat. 4. Check inhaler technique: Ensure that you're using the inhaler correctly to minimize the amount of medicine that can deposit in your mouth. If you have developed white patches and a bad taste, it's important to contact your healthcare provider. They can diagnose the issue, potentially prescribe antifungal medication if it's thrush, and give you specific advice on how to manage these side effects while continuing to treat your asthma.
15. I was recently diagnosed with hypertension and prescribed a drug called captopril. I heard that potassium supplements can help lower blood pressure, is this true?	Potassium is an essential mineral that plays a significant role in maintaining proper heart function and regulating blood pressure. It's true that potassium can help lower blood pressure for some people, particularly because it lessens the effects of sodium in the diet and helps to ease tension in the blood vessel walls. However, when it comes to taking potassium supplements, especially when you are on blood pressure medication like captopril (which is an ACE inhibitor), it is very important to proceed with caution. ACE inhibitors can increase the level of potassium in your blood, and too much potassium can be harmful. Hyperkalemia, or high potassium levels, can lead to serious heart problems and should be avoided. Here are some key points to consider: 1. Consult Your Doctor: Always talk to your healthcare provider before starting any new supplements, including potassium. They can provide advice based on your specific health needs and medications. 2. Monitor Potassium Levels: If your doctor agrees that you should increase your potassium intake, they may recommend dietary changes instead of supplements and will monitor your potassium levels. 3. Dietary Sources: Potassium is abundant in many foods, such as bananas, oranges, potatoes, spinach, and beans. A diet rich in these foods may be a safer way to increase your potassium intake compared to supplements. 4. Regular Check-Ups: It's important to have regular blood tests to monitor your potassium levels if you are on captopril, as your kidneys' ability to manage potassium is affected by this medication. If you have hypertension, lifestyle changes such as a balanced diet, regular exercise, maintaining a healthy weight, and managing stress are important in managing your blood pressure. Always follow the treatment plan prescribed by your healthcare provider.
16. I am a 45 years old female and I am prescribed lithium to help treat my mood swings. My husband is used to taking a drug called furosemide to treat his feet that keep swelling. Recently, my feet started swelling as well and I would like to ask if I can also use furosemide?	Furosemide is a potent diuretic (water pill) that is used to treat edema (swelling) associated with congestive heart failure, liver disease, or kidney disease, and to manage high blood pressure. It's important to note that furosemide is a prescription medication, and its use needs to be closely monitored by a healthcare provider due to potential side effects and interactions with other medications. As for your situation, taking furosemide while on lithium requires careful consideration: 1. Lithium Interaction: Furosemide can affect lithium levels in the body, potentially leading to lithium toxicity, which is a serious risk. Lithium levels need to be closely monitored if a diuretic like furosemide is added to your medication regimen. 2. Underlying Causes: Swelling of the feet can have many causes, and it is important to determine the underlying reason for your symptoms before starting treatment. The cause of edema in your case may be different from that of your husband. 3. Medical Advice Needed: You should not take furosemide without a prescription and without consulting your healthcare provider. They will need to evaluate your condition to determine the cause of your swelling and to decide whether furosemide is appropriate for you. 4. Monitoring and Tests: If your doctor prescribes furosemide, they will monitor your kidney function, electrolytes, and lithium levels regularly. It's crucial to get personalized medical advice from your healthcare provider. Self-medicating, especially when it involves prescription medications like furosemide and lithium, can be dangerous. Please contact your healthcare provider to discuss your symptoms and the best course of action.
17. I am a 25 years old female and I suffer from epilepsy but it is controlled using Lamotrigine, I recently got married and I am planning to become pregnant. Is my drug safe in pregnancy?	Lamotrigine is an antiepileptic drug that is used to control seizures. The safety of lamotrigine during pregnancy is an important consideration, as some antiepileptic drugs can have risks for both the mother and the developing fetus. Here’s what is generally known about lamotrigine and pregnancy: Risks vs. Benefits: The use of lamotrigine during pregnancy may be advised if the benefits to the mother outweigh the potential risks to the fetus. Uncontrolled seizures in pregnancy can have serious consequences for both the mother and the unborn baby. Lower Risk Category: Lamotrigine is one of the antiepileptic drugs that is considered to have a lower risk of birth defects compared to some other medications used for epilepsy. However, no drug can be considered completely safe. Monitoring: If you are taking lamotrigine and planning to become pregnant, it is important to discuss this with your healthcare provider. They may adjust your medication dose and will likely recommend more frequent monitoring. Folic Acid: Women on antiepileptic drugs are often advised to take folic acid supplements before conception and during pregnancy to decrease the risk of neural tube defects. Registry: Pregnant women on antiepileptic drugs, including lamotrigine, are often encouraged to register with a pregnancy registry that tracks outcomes. This can provide valuable information for other women and healthcare providers. It's essential that you do not stop taking your medication without consulting your healthcare provider, as having a seizure during pregnancy could harm both you and your baby. Your healthcare provider will work with you to manage your condition in a way that is safest for your pregnancy. Please consult with your healthcare provider or a specialist in maternal-fetal medicine to discuss the best approach to manage your epilepsy before and during pregnancy. They can provide guidance tailored to your personal health needs.
18. I am a breastfeeding 30 years old female and I am considering taking a contraceptive, is there an effective option that would be safe for me and my baby?	While breastfeeding, it's important to choose a contraceptive method that is safe for both you and your baby. Certain hormonal contraceptives can affect milk supply, so options are generally considered based on their potential impact on lactation and the baby. The following contraceptive options are typically considered safe during breastfeeding: 1. Progestin-only contraceptives: These include pills (often called the mini-pill), the injectable contraceptive (such as Depo-Provera), implants (like Nexplanon), and intrauterine devices (IUDs). They do not contain estrogen, which is the hormone most likely to interfere with milk supply. 2. Intrauterine Devices (IUDs): Both hormonal and non-hormonal (copper) IUDs are effective and can be used while breastfeeding. 3. Barrier methods: Condoms, diaphragms, and cervical caps are also safe to use during breastfeeding and do not interfere with milk supply or pass any hormones to the baby. 4. Natural family planning methods: Methods that involve monitoring fertility signs to avoid sex during fertile times can be less reliable during breastfeeding due to the natural changes in menstrual cycles. It's important to discuss with your healthcare provider which method would be the best for your specific situation. They can provide advice based on your health history, breastfeeding status, and any other relevant factors. They can also ensure that you understand how to use the method effectively and discuss any potential side effects.
19. my 6 months old baby is feeling warm, his left cheek is reddish and he keeps rubbing his ear. Is he ok or is there something wrong?	If your baby is feeling warm, has a reddish cheek, and is rubbing his ear, it's possible that he may be experiencing discomfort or pain related to an ear infection or teething. Ear infections are common in young children and can cause symptoms such as fever, fussiness, trouble sleeping, and pulling or rubbing at the ears. Teething can also cause similar symptoms, including red cheeks and ear rubbing, as the pain in the gums can radiate to the ears. Here's what you can do: 1. Check Temperature: Use a thermometer to check if your baby has a fever. A temperature above 100.4°F (38°C) is considered a fever in infants. 2. Look for Other Symptoms: Watch for other signs of illness, such as irritability, changes in sleeping or eating patterns, or a runny nose, which might suggest an infection. 3. Teething Signs: If teething is the cause, you might also notice drooling, a desire to chew on hard objects, and swollen gums. 4. Consult Healthcare Provider: Given that your baby is only 6 months old and is exhibiting these symptoms, it's important to contact your pediatrician or healthcare provider for an accurate diagnosis and appropriate treatment. In the meantime, ensure that your baby is comfortable, well-hydrated, and if he is feverish, dressed in light clothing to prevent overheating. Avoid putting anything in the baby’s ear (like ear drops or oils) unless advised by a healthcare provider. If at any point your baby seems to be in significant discomfort, has a high fever, or if you notice any other concerning symptoms, seek medical attention promptly.
20. my 12 months old baby is continuously coughing and I have some spare SINECOD syrup, is it ok if I give it to him?	Sinecod (which contains butamirate) is a cough suppressant that is used to treat coughs of various origins. However, it's important to exercise caution when administering any medication to a baby or young child. For infants or children, especially those under the age of 2 years, it is critical not to use over-the-counter cough and cold products unless instructed by a healthcare provider. The American Academy of Pediatrics advises against the use of cough and cold preparations in young children due to potential risks and side effects, which can be serious. Here are a few guidelines: 1. Consult a Pediatrician: Always consult with a pediatrician before giving any medication, including cough syrup, to your baby. 2. Dosage and Safety: If a healthcare provider deems it necessary to use such a medication, they will provide the correct dosage based on the baby's weight and age. Dosages for infants and children are not the same as for adults. 3. Symptom Monitoring: Monitor other symptoms that your baby may have, such as fever, difficulty breathing, or changes in eating or sleeping patterns. 4. Immediate Care: If your baby's cough is severe, if they are having trouble breathing, or if they show any signs of distress, seek immediate medical attention. It's important to accurately diagnose the cause of the cough and to treat any underlying conditions. Please contact your child's pediatrician for an evaluation and for advice on how to manage the cough.
ChatGPT 4.0 Trial 2 Responses	
Question	Response
1. how should I store my LANTUS and NOVOLOG insulin pens?	For Lantus (insulin glargine) and NovoLog (insulin aspart) insulin pens, storage guidelines are important to maintain the effectiveness of the insulin. Here's how you should store them: 1. Before First Use: Store new, unused insulin pens in the refrigerator at 36°F to 46°F (2°C to 8°C). Do not freeze them. If they freeze, discard the pens as freezing can damage insulin. 2. After First Use: Once in use, you can keep the pens at room temperature, below 86°F (30°C), for the number of days indicated by the manufacturer. For Lantus, it's typically 28 days, and for NovoLog, it's 28 days as well. Keep the pen away from direct heat and light. The pen cap should be put back on the pen after each injection to protect from light. 3. Do Not: Do not use the insulin after the expiration date printed on the pen or box. Do not store the pen with the needle attached, as it may lead to leakage, blockage, or contamination. 4. Travel: When traveling, keep your insulin pens in a cool bag if you expect to be in a hot environment or if you are unable to refrigerate them. Remember to always check the appearance of your insulin. It should be clear and colorless. Do not use the insulin if it looks cloudy, thickened, or colored, or if it has solid particles. For the most accurate storage instructions, please refer to the information that came with your medication or consult with your healthcare provider.
2. how many times per day should I use albuterol for my asthma?	Albuterol is a medication used to treat and prevent bronchospasm in people with asthma or certain other airway diseases. The dosage and frequency of albuterol use can vary depending on the individual's treatment plan and the severity of symptoms. Here are general guidelines for albuterol use in asthma management: 1. Rescue Treatment: Albuterol is often prescribed as a rescue inhaler, which means it is used to provide quick relief from acute asthma symptoms. As a rescue inhaler, it can be used every 4 to 6 hours as needed for symptom relief. 2. Scheduled Doses: Some individuals may be advised to use it on a regular schedule, which is less common and typically occurs under specific medical advice when asthma symptoms are not well controlled. 3. Exercise-Induced Asthma: For preventing exercise-induced bronchospasm, albuterol is typically used about 15 to 30 minutes before exercise. It's important not to use albuterol more frequently than recommended by your healthcare provider. Overuse can lead to decreased effectiveness and increased side effects. Always follow the dosing instructions provided by your healthcare provider or the directions on the prescription label. If you find that you need to use albuterol more often than prescribed, or if it's not as effective at relieving your asthma symptoms, you should contact your healthcare provider as this may be a sign that your asthma is not well controlled.
3. can you provide step by step instructions on how to take CLEXANE 40mg pre-filled syringes?	Certainly, Clexane (known as Enoxaparin in the United States) is an anticoagulant medication used to prevent and treat blood clots. Here is a step-by-step guide on how to use Clexane 40 mg pre-filled syringes: 1. Preparation: Wash your hands thoroughly with soap and water. Choose a clean, well-lit working area. Lay out all the materials needed: the Clexane syringe, alcohol swabs, and a sharps disposal container. 2. Syringe Inspection: Check the expiration date on the syringe and ensure the solution is clear and particle-free. Do not expel the air bubble in the syringe before the injection unless instructed by your healthcare provider, as it is meant to be there. 3. Site Selection: The recommended injection sites are the left and right sides of the abdomen, at least 2 inches away from the belly button. Alternate the injection site with each dose to reduce soreness. 4. Cleaning the Injection Site: Use an alcohol swab to clean the area where the injection will be given. Allow the skin to air dry. 5. Injection Technique: Remove the needle cover from the syringe. Pinch a fold of skin between your thumb and forefinger. Hold the syringe like a pencil and insert the needle into the skin fold at a 45 to 90-degree angle. Once the needle is fully inserted, press down on the plunger to inject the medication. Leave the needle in the skin for a few seconds to ensure all the medication has been delivered. 6. After Injection: Do not rub the injection site. You may gently press a cotton ball or gauze onto the site if there is any bleeding. Dispose of the syringe in a sharps disposal container immediately after use. Do not reuse the syringe or needle. 7. Post-Injection Care: If you notice any redness, swelling, or pain at the injection site, or if you experience unusual bleeding or bruising, contact your healthcare provider. Please follow the specific instructions given to you by your healthcare provider, as they may have specific instructions tailored to your condition. If you are unsure about any step or have difficulty with the injections, contact your healthcare provider for assistance or a demonstration.
4. should I take aspirin 100mg with food or on an empty stomach?	Aspirin, particularly in low doses such as 100mg which is often used for its antiplatelet effects, should generally be taken with food or a full glass of water. Taking aspirin with food or after a meal can help minimize gastric irritation and protect the stomach lining, as aspirin can be harsh on the stomach and may cause irritation or ulcers in some individuals. Always follow the advice of your healthcare provider regarding how to take your medications, as they may have specific recommendations for your situation. If you have been prescribed aspirin and have not been given specific instructions on how to take it, or if you experience any stomach discomfort, it's a good idea to consult with your healthcare provider or pharmacist for personalized advice.
5. can you advise me on how to safely dispose my insulin syringes?	Certainly! Disposing of insulin syringes safely is important to prevent needle-stick injuries and environmental contamination. Here's how to do it: 1. Use a Sharps Disposal Container: Place used syringes in a FDA-cleared sharps disposal container immediately after use. If an FDA-cleared container is not available, you can use a heavy-duty plastic household container with a tight-fitting lid, such as a laundry detergent container. 2. Proper Container: Your container should be leak-resistant, remain upright during use, and be properly labeled to warn of hazardous waste inside the container. 3. Do Not Recycle: Do not throw loose needles and syringes into the trash or recycling bins, and never flush them down the toilet. 4. When the Container is Full: When your sharps disposal container is almost full, follow your community guidelines for proper disposal methods. Some options for disposal include bringing the container to a drop-off collection site like a pharmacy, hospital, or doctor’s office, utilizing a mail-back program, or using a residential special waste pick-up service if available in your area. 5. Check Local Guidelines: Rules and guidelines for disposal vary by location, so check with your local waste management authorities for specific instructions. 6. Keep Out of Reach: Until you can dispose of the full container, keep it out of reach of children and pets. Never try to remove, bend, break, or recap needles used by another person. This can lead to accidental needle sticks, which may cause serious infections. It's great that you're taking the initiative to dispose of your medical waste responsibly!
6. I can’t remember if I should take LOZOL in the morning or at night, can you advise me?	Lozol, which is the brand name for Indapamide, is a diuretic medication often used to treat high blood pressure and swelling due to fluid buildup. It works by causing you to make more urine, which helps your body get rid of extra salt and water. Indapamide is usually advised to be taken in the morning. Taking it in the morning helps reduce the likelihood of needing to get up in the night to urinate, which can disrupt sleep. However, your healthcare provider's instructions about when to take it should take precedence. If you've been given specific advice from your healthcare provider, you should follow that. If you don't have specific instructions or if you're still unsure, you should contact your healthcare provider or pharmacist to clarify when to take your medication to ensure it is effective and to minimize side effects.
7. I took an ibuprofen 600mg pill for a headache 1 hour ago but it’s not working, should I take another one?	It's important to use caution when taking medications like ibuprofen. Ibuprofen 600 mg is a prescription-strength dose, and it is generally recommended to take it every 6 to 8 hours as needed, with a maximum of three doses in 24 hours. If you've taken 600 mg of ibuprofen and it hasn't relieved your headache after 1 hour, it's best not to take an additional dose yet. It can take longer for the full effects of ibuprofen to be felt, sometimes up to several hours. Taking another dose too soon could increase the risk of side effects. Here are some steps you can take: 1. Wait a bit longer: Give it a little more time to see if the headache subsides. 2. Consider other factors: Ensure you're hydrated, have eaten, and are in a comfortable environment as these can also affect headache severity. 3. Use adjunct therapy: Sometimes, a cold pack or resting in a dark room can also help alleviate headache symptoms. 4. Consult a healthcare professional: If the headache persists or worsens, or if you frequently experience headaches, it would be wise to consult with a healthcare provider. 5. Read the label: Always follow the dosage instructions on the medicine label or those provided by your healthcare provider. Remember, it's crucial to follow dosing instructions and to consult with a healthcare professional before exceeding the recommended dose. If you have any underlying health conditions or are taking other medications, it's especially important to speak with a healthcare provider before taking additional doses of any medication.
8. I usually take my ATACAND pill every morning but I don’t remember if I took it today, what should I do?	Forgetting to take a dose of medication like Atacand (candesartan) can happen, and there are general guidelines for what to do in such situations. However, it's always best to follow the specific advice given by your healthcare provider or the instructions that came with your medication. Here's what is typically recommended: 1. If You Remember Shortly After Your Usual Time: If it's only been a short while since the time you usually take Atacand, take the missed dose as soon as you remember. 2. If It's Closer to the Time of Your Next Dose: If it's almost time for your next scheduled dose, skip the missed dose. Do not double up to make up for the missed dose. 3. Maintain Your Regular Schedule: Continue with your regular dosing schedule the next day. 4. Double Doses: Never take a double dose to make up for a forgotten one. 5. Use a Reminder System: To help remember your doses, use a medication box with days of the week, set alarms, or use a medication tracking app. 6. Consult Your Healthcare Provider: If you're unsure or if missing doses happens frequently, consult your healthcare provider for more personalized advice. It's important to take your blood pressure medication consistently to maintain its effectiveness. If you miss doses often, it might be worth discussing this with your healthcare provider to see if there's a way to make it easier to remember your medication, or to check if any adjustments need to be made to your treatment plan.
9. I always take my metformin tablet at the same time every day but I overslept and missed the dose by 3 hours, what should I do?	If you've missed your dose of metformin by 3 hours, the general advice is to take the missed dose as soon as you remember, as long as it's not too close to the time of your next scheduled dose. If your next dose is due within a few hours, you should skip the missed dose to avoid doubling up. Then continue with your regular dosing schedule. Metformin is usually prescribed to be taken with meals to help reduce stomach or bowel side effects that may occur during the first few weeks of treatment. So if it's still around mealtime when you remember, you can take it with food. Here are steps you can follow: 1. Take the Missed Dose: If it's only been 3 hours, it's generally safe to take your missed dose, particularly if you have a meal around that time. 2. Skip if Close to Next Dose: If it's close to the time for your next dose, just take the next dose at your regular time. 3. Do Not Double Up: Never take a double dose to make up for a dose you have missed. 4. Monitor Blood Sugar: Keep an eye on your blood sugar levels to ensure they remain stable. 5. Set Reminders: Consider setting an alarm or reminder for your medication to prevent missing doses in the future. 6. Consult Your Healthcare Provider: If you're unsure or if you miss doses often, speak to your healthcare provider for personalized advice. Remember, these are general guidelines, and it's always best to follow the specific advice given by your healthcare provider.
10. I was prescribed FOSAMAX 70 mg tablets to take once weekly for my osteoporosis and my physician gave me an instructions flyer on how to take the drug but I lost it, are there any important use instructions?	Fosamax (alendronate sodium) is a bisphosphonate used to treat and prevent osteoporosis. It is important to follow specific instructions when taking Fosamax to ensure effectiveness and minimize side effects. Here are the general instructions: 1. Time of Day: Take Fosamax first thing in the morning, at least 30 minutes before you eat or drink anything or take any other medications. 2. Before Eating or Drinking: Take it on an empty stomach with a full glass of plain water (6-8 ounces) to facilitate delivery to the stomach and minimize the potential for esophageal irritation. 3. Do Not Lie Down: After taking Fosamax, do not lie down, recline, or go to bed for at least 30 minutes, and not until after you eat your first food of the day. This helps prevent the medication from irritating the esophagus. 4. Swallow Whole: Swallow the tablet whole. Do not chew or suck on it, and do not allow it to dissolve in your mouth. 5. Other Beverages: Do not take Fosamax with mineral water, coffee, juice, or any liquid other than plain water. 6. Missed Dose: If you miss a dose, take it the morning after you remember. Then return to your original once-weekly schedule on the chosen dose day. Do not take two doses on the same day. 7. Calcium and Vitamins: Wait at least 30 minutes after taking Fosamax before taking any other medication, including antacids, calcium, and vitamins. 8. Special Instructions: Follow any other specific instructions your physician may have given you regarding your overall health condition. 9. Side Effects and Concerns: Contact your healthcare provider if you experience difficulty or pain upon swallowing, chest pain, new or worsening heartburn, or if you have pain in your hips, groin, or thighs as these could be signs of a serious condition. 10. Regular Dental Check-ups: It's important to maintain good oral hygiene and get regular dental check-ups, as bisphosphonates have been associated with dental issues like osteonecrosis of the jaw. If you've lost the flyer with the specific instructions from your physician, you might want to contact your doctor's office to get a replacement or to confirm that these general instructions apply to your particular situation.
11. what is the drug Glyburide used for and how does it work?	Glyburide, also known as glibenclamide in some countries, is an oral diabetes medication used to control blood sugar levels in people with type 2 diabetes. It is part of a class of diabetes drugs called sulfonylureas. Here's how it works: 1. Insulin Secretion: Glyburide works by stimulating the beta cells in the pancreas to release insulin. This process requires functional pancreatic beta cells, which is why glyburide is not used in type 1 diabetes, where the pancreatic beta cells are not able to produce insulin. 2. Increased Insulin Sensitivity: It may also help the body's cells use insulin more effectively by increasing their sensitivity to insulin. 3. Lower Blood Sugar: By increasing insulin release and improving the effectiveness of insulin, glyburide helps lower blood sugar levels. It is important to note that glyburide is typically used when diet, exercise, and other lifestyle modifications have not been enough to control blood glucose. It can be used alone or with other medications such as metformin. As with any medication, there are potential side effects and risks, including the risk of hypoglycemia (low blood sugar), especially if meals are skipped or delayed, heavy exercise is not compensated by caloric intake, or when combined with other anti-diabetic drugs. Patients taking glyburide should monitor their blood sugar regularly and be aware of the signs and symptoms of hypoglycemia, which can include dizziness, shaking, sweating, or hunger. It's also important to maintain a consistent eating schedule and not to consume alcohol excessively, as it can increase the risk of hypoglycemia. If you have been prescribed glyburide, be sure to follow your healthcare provider's instructions closely, and discuss any concerns or questions you may have with them.
12. my physician told me about one of the drugs I take to use it only when I feel chest pain or before exercise but I cannot remember which one, I take the following medications: CONCOR, CRESTOR and NITROSTAT, can you tell me which one is it and how does it work?	The medication from your list that is used as needed for chest pain or before exercise to prevent chest pain is Nitrostat, which is a brand name for nitroglycerin. Nitroglycerin is a nitrate that works by relaxing and widening blood vessels, which allows more blood to flow to the heart while reducing its workload. When you feel chest pain (angina), taking Nitrostat can help alleviate the pain by improving blood flow. It's also used prophylactically before activities that might cause chest pain due to increased demand on the heart. Here's how to use Nitrostat for chest pain: 1. At the Onset of Pain: Take Nitrostat at the first sign of chest pain. Sit down when you use the tablets. 2. Under the Tongue: Place a tablet under your tongue or in the cheek pouch and allow it to dissolve. Do not chew or swallow the tablet. 3. Wait 5 Minutes: If the pain persists after 5 minutes, you can take a second tablet. 4. Call for Help: If the pain continues after three tablets in a 15-minute period, seek emergency medical attention. Nitrostat is typically used only when needed, as opposed to Concor (a beta-blocker) and Crestor (a statin), which are usually taken on a regular schedule. Concor is used to manage hypertension and heart rhythm disorders, while Crestor is used to manage cholesterol levels. It's very important to follow your physician's instructions on how to use these medications properly, and to understand which medications are to be taken on a regular schedule and which are to be taken as needed. If there is any uncertainty, you should confirm with your healthcare provider or pharmacist.
13. my son was prescribed ZINNAT 250mg and I forgot to mention to the doctor that my son is allergic to penicillin, is it safe for him to take it?	Zinnat, which contains the active ingredient cefuroxime, is a type of antibiotic known as a cephalosporin. Cephalosporins are chemically related to penicillin. In individuals who have a penicillin allergy, there is a known cross-reactivity risk between penicillins and cephalosporins, although this risk is generally low. However, if your son has a history of a severe allergic reaction to penicillin (such as anaphylaxis), then it is generally advised to avoid cephalosporins unless an allergist has evaluated the risk and determined it to be safe. The exact risk can vary depending on the specific cephalosporin and the nature of the penicillin allergy. Because of your son's allergy, you should contact the prescribing physician immediately to discuss this issue before administering Zinnat. The doctor may choose to prescribe a different antibiotic that does not carry the same risk of cross-reactivity. Do not give the medication to your son until you have spoken with the healthcare provider.
14. I am an asthmatic patient who was recently prescribed FLIXOTIDE EVOHALER, I noticed the appearance of white patches on my tongue and I feel a very bad taste. I don’t know if this is because of the drug or asthma, can you help?	The symptoms you're describing — white patches on the tongue and a bad taste in the mouth — could be due to a condition known as oral thrush, which is a yeast infection in the mouth. Oral thrush can sometimes occur in people using inhaled corticosteroids, like Flixotide (fluticasone propionate), for asthma. Inhaled steroids can affect the immune response in your mouth and throat, which can lead to an overgrowth of the fungus Candida, causing thrush. It's not directly related to asthma itself, but rather a potential side effect of the treatment. To help prevent oral thrush when using an inhaler, you can: 1. Rinse Your Mouth: After using the inhaler, rinse your mouth with water, gargle, and spit out the water without swallowing it. 2. Use a Spacer: If you're not already doing so, use a spacer device with your inhaler, as this can help reduce the amount of medication that deposits in your mouth and throat. 3. Good Oral Hygiene: Maintain good oral hygiene, including regular brushing and flossing. If you've developed white patches and a bad taste, it's advisable to contact your healthcare provider. They can confirm whether it's oral thrush and may prescribe antifungal medication to treat the infection. It's also important to not stop your asthma medication without consulting your healthcare provider, as they can give you the best advice on managing both your asthma and any side effects from the treatment.
15. I was recently diagnosed with hypertension and prescribed a drug called captopril. I heard that potassium supplements can help lower blood pressure, is this true?	Potassium is a mineral that plays several critical roles in the body, including the regulation of heart function and blood pressure. Indeed, potassium can help lower blood pressure by balancing out the negative effects of salt and by helping to relax blood vessel walls. However, while increasing dietary potassium can be beneficial for most people with high blood pressure, potassium supplements are not appropriate for everyone and should be taken cautiously, especially if you're on certain blood pressure medications, like captopril. Captopril is an ACE inhibitor, which can increase potassium levels in the blood. Taking potassium supplements along with ACE inhibitors can lead to too much potassium in the blood, a condition known as hyperkalemia, which can be dangerous and potentially life-threatening. Before considering potassium supplements, it's important to discuss with your healthcare provider, as they can assess your current potassium levels and overall health status. They might recommend increasing potassium through diet instead of supplements. Foods rich in potassium include bananas, oranges, potatoes, spinach, and beans. If your doctor advises increasing your potassium intake through your diet, it's usually a safe and healthy way to potentially help manage your blood pressure. But if you're considering potassium supplements, it's essential to do this under medical supervision to avoid potential complications.
16. I am a 45 years old female and I am prescribed lithium to help treat my mood swings. My husband is used to taking a drug called furosemide to treat his feet that keep swelling. Recently, my feet started swelling as well and I would like to ask if I can also use furosemide?	It's important to be cautious when considering taking a medication prescribed to someone else, even if the symptoms seem similar. Furosemide is a potent diuretic (often known as a "water pill") used to treat edema (swelling) and fluid retention caused by various medical conditions such as heart failure, liver disease, or kidney disease. Regarding your situation with lithium and the swelling in your feet, there are a few considerations: Lithium and Diuretics: Lithium levels can be affected by changes in sodium and fluid levels in the body. Diuretics like furosemide can increase lithium levels, potentially leading to toxicity. This is a serious risk and requires close monitoring by a healthcare professional. Causes of Swelling: Swelling can be caused by a variety of factors and may not always be appropriate to treat with a diuretic. It is important to determine the cause of the swelling before starting treatment. Self-Medication Risks: Self-medicating with furosemide without a doctor's guidance can lead to significant health risks, including dehydration, electrolyte imbalances, and potentially affecting the levels and effectiveness of other medications you are taking. Given these points, it is crucial that you do not take your husband's furosemide and instead consult with your healthcare provider. They will be able to assess the cause of your swelling and recommend the appropriate treatment. This may include investigating the dose and effects of your current lithium treatment or considering other causes for the swelling.
17. I am a 25 years old female and I suffer from epilepsy but it is controlled using Lamotrigine, I recently got married and I am planning to become pregnant. Is my drug safe in pregnancy?	Lamotrigine is an antiepileptic drug that is sometimes used during pregnancy to control seizures. When it comes to using lamotrigine during pregnancy, there are a few important considerations: 1. Risk of Seizures: Uncontrolled seizures can be harmful to both the mother and the unborn baby, so it's important to continue seizure medication during pregnancy. 2. Risks of Medication: While lamotrigine is one of the antiepileptic drugs that are considered relatively safe during pregnancy, there is still a risk of birth defects, as with many medications. 3. Dosage Adjustments: The metabolism of lamotrigine can change during pregnancy, and dosage adjustments may be necessary to maintain therapeutic levels and control seizures. 4. Folic Acid: Women on antiepileptic drugs are often advised to take folic acid supplements before conception and during pregnancy to reduce the risk of neural tube defects in the baby. 5. Pregnancy Registry: Pregnant women who take antiepileptic medications are encouraged to enroll in pregnancy registries, which monitor the health of women and their babies to gather more information about the safety of these medications during pregnancy. It's crucial to discuss your plans to become pregnant with your healthcare provider, preferably before conception. Your doctor can give you the most current information on the risks and benefits of using lamotrigine during pregnancy and can help you weigh those against the risks of uncontrolled epilepsy. Your medication regimen may need to be adjusted, and your pregnancy will likely be closely monitored. Do not stop taking your medication without consulting your healthcare provider, as this could lead to a return of seizures, which could be harmful. Your healthcare provider will work with you to find the safest and most effective treatment plan during your pregnancy.
18. I am a breastfeeding 30 years old female and I am considering taking a contraceptive, is there an effective option that would be safe for me and my baby?	When considering birth control options while breastfeeding, it's important to choose methods that are safe for both you and your baby and that won't affect your milk supply. Here are some contraceptive options that are generally considered safe for breastfeeding mothers: 1. Progestin-only Pills (POPs): Also known as the "mini-pill," these contain only progestin and no estrogen, which is generally preferred during breastfeeding because it's less likely to affect milk production. 2. Intrauterine Devices (IUDs): Both hormonal and non-hormonal (copper) IUDs are safe to use while breastfeeding and do not affect milk supply. 3. Implants: Progestin-only implants, such as Nexplanon, are safe to use while breastfeeding. 4. Injections: The Depo-Provera shot, a progestin-only injection given every three months, is also considered safe for breastfeeding mothers. 5. Barrier Methods: Methods such as condoms, diaphragms, and cervical caps can be used anytime during breastfeeding. 6. Natural Family Planning: This involves tracking fertility signs to avoid sex during fertile periods. While it doesn't involve hormones, its effectiveness can be variable. Estrogen-containing contraceptives, like combined oral contraceptives (the regular birth control pill), are usually not recommended for breastfeeding mothers, especially in the first few weeks postpartum, as they can affect milk supply. It's important to consult with your healthcare provider to discuss which option would be best for you, considering your health history, breastfeeding situation, and personal preference. Your healthcare provider can give you the most appropriate advice and ensure that your contraceptive choice is safe, effective, and suitable for your situation.
19. my 6 months old baby is feeling warm, his left cheek is reddish and he keeps rubbing his ear. Is he ok or is there something wrong?	The symptoms you're describing — warmth, a reddish cheek, and rubbing the ear — could suggest that your baby may have an ear infection, which is quite common in children. Ear infections can cause discomfort and pain, which might make a baby rub or pull at their ear. Fever and redness can also be associated symptoms. However, it's important to note that babies at this age can also exhibit these signs due to teething. But since an accurate diagnosis is important and I cannot provide one, it's best to have your baby seen by a pediatrician. The pediatrician will be able to examine your baby's ear, assess for any signs of infection or other issues, and provide appropriate care. If your baby seems to be in discomfort, is running a fever, or if you have any concerns about his health, it's best to err on the side of caution and contact your pediatric healthcare provider for advice as soon as possible.
20. my 12 months old baby is continuously coughing and I have some spare SINECOD syrup, is it ok if I give it to him?	Sinecod (which contains butamirate) is a cough medicine that's used to suppress coughing. It's very important to use caution when giving any medication to a baby, as the dosages and potential side effects can be quite different than for older children or adults. For infants and young children, especially those under the age of 2, it's generally recommended to avoid over-the-counter cough and cold medications unless advised by a healthcare professional. This is because of the potential for serious side effects and the risk of overdose. Before administering Sinecod or any medication to your 12-month-old baby, you should consult with a pediatrician or healthcare provider to ensure it's safe and appropriate for their age, weight, and symptoms. The pediatrician may need to evaluate the baby to determine the cause of the cough and the best treatment. Do not give the medication without professional medical advice. If your baby is having difficulty breathing, is not drinking or eating, or if you have any other concerns, seek medical attention promptly.
